# Combined Effect of Per- and Polyfluoroalkyl Substances, Toxic Metals, Phthalates and Volatile Organic Compounds on Reproductive Hormones

**DOI:** 10.3390/pollutants6020031

**Published:** 2026-06-15

**Authors:** Issah Haruna, Emmanuel Obeng-Gyasi

**Affiliations:** 1 Department of Built Environment, North Carolina A&T State University, Greensboro, NC 27411, USA; 2 Environmental Health and Disease Laboratory, North Carolina A&T State University, Greensboro, NC 27411, USA

**Keywords:** endocrine-disrupting chemicals, chemical mixtures, reproductive hormones, Bayesian Kernel Machine Regression, NHANES

## Abstract

**Background:**

Human exposure to environmental endocrine-disrupting chemicals (EDCs) rarely occurs in isolation, yet most epidemiological research has assessed chemicals individually. PFASs, toxic metals, phthalates, and VOCs are ubiquitous contaminants with well-documented reproductive toxicity.

**Objective:**

The aim of this study was to investigate the joint and individual effects of 28 EDCs spanning four chemical classes on six reproductive hormone biomarkers in a nationally representative U.S. population—using an innovative approach that simultaneously characterizes nonlinear mixture effects and chemical interactions across multiple exposure domains.

**Methods:**

This cross-sectional study used NHANES 2017–2018 data (*n* = 9254). Multivariable linear regression and Bayesian Kernel Machine Regression (BKMR) characterized individual and mixture associations, respectively. Missing data were handled using multiple imputations by chained equations. Survey design weights were applied in linear regression models.

**Results:**

Linear regression revealed heterogeneous associations across chemical classes and hormones. PFOA was positively associated with SHBG (β = 12.35; 95% CI: 8.33, 16.38) and LH (β = 6.91; 95% CI: 1.44, 12.38), while mercury was inversely associated with estradiol (β = −3.38; 95% CI: −5.12, −1.65). BKMR analyses identified pronounced non-monotonic dose–response relationships and emergent mixture effects not predictable from single-chemical analyses for all six hormones. Posterior inclusion probabilities identified cadmium, PFOA, MEHP, and MBzP as the most influential predictors across hormone endpoints.

**Conclusions:**

Concurrent real-world exposure to PFASs, toxic metals, phthalates, and VOCs is associated with measurable, nonlinear alterations in reproductive hormone profiles. Chemical mixture effects cannot be reliably predicted from single-pollutant analyses, underscoring the necessity of mixture-based methodologies in environmental reproductive epidemiology. Prospective studies are needed to establish causal temporality and identify critical windows of susceptibility.

## 1. Introduction

### 1.1 Environmental Impact on Reproductive Hormones

The human reproductive system is a complex network of hormonal interactions that regulate fertility, sexual development, and numerous physiological processes throughout our lifespan [1]. Reproductive hormones, including estradiol, follicle-stimulating hormone (FSH), luteinizing hormone (LH), progesterone, sex-hormone-binding globulin (SHBG), and anti-Müllerian hormone (AMH), play critical roles in maintaining reproductive health in both males and females [2,3]. These hormones orchestrate essential functions ranging from gamete production and sexual maturation to pregnancy maintenance and metabolic regulation [2]. However, this intricate endocrine system is increasingly vulnerable to disruption by environmental chemicals, raising significant concerns about reproductive health outcomes in the exposed population [4,5].

Environmental endocrine-disrupting chemicals (EDCs) represent a diverse group of synthetic and naturally occurring compounds that can interfere with hormone synthesis, transport, metabolism, or action [6]. The widespread presence of EDCs in consumer products, industrial processes, and environmental media has resulted in ubiquitous human exposure, with detectable levels found in virtually all individuals tested in biomonitoring studies [7]. Among the most concerning classes of EDC_S_ are per- and polyfluoroalkyl substances (PFASs), toxic metals, phthalates, and volatile organic compounds (VOCs), each of which has been associated with reproductive hormone alteration and adverse reproductive outcomes.

### 1.2. Reproductive Hormones and Endocrine Disruptions

The hypothalamic–pituitary–gonadal (HPG) axis is the primary regulatory system for reproductive hormone production and function [8]. This axis involves complex feedback mechanisms between the hypothalamus, anterior pituitary, and gonads, which can be disrupted at multiple levels by environmental chemicals [4]. Sex-hormone-binding globulin (SHBG), a hepatic glycoprotein, regulates the bioavailability of circulating sex steroids and serves as a sensitive marker of hepatic responses to endocrine disruption [9]. Progesterone, synthesized primarily in the corpus luteum, is essential for luteal phase maintenance, implantation, and pregnancy, and is a key indicator of ovulatory function [10]. In females, estradiol regulates the menstrual cycle, supports pregnancy, and influences bone health and cardiovascular function [11]. Gonadotropins, including FSH and LH, stimulate gonadal function and are critical for normal reproductive physiology in both sexes [12].

Endocrine disruption can occur through various mechanisms, including interference with hormone receptors, alteration of enzyme activities involved in hormone synthesis and metabolism, and disruption of hormone transport proteins [13,14]. The timing of exposure is particularly crucial as reproductive hormones play an essential role during critical development windows, including fetal development, puberty, and reproductive maturity. Exposure during these sensitive periods can result in permanent alteration of reproductive function and increased susceptibility to reproductive disorders later in life [15].

### 1.3. Per- and Polyfluoroalkyl Substances (PFASs)

PFASs comprise a large class of synthetic chemicals characterized by their strong carbon–fluorine bonds, which confer exceptional chemical stability and resistance to degradation [16,17]. This persistence has earned PFASs a designation as “forever chemicals”, as they accumulate in the environment and human tissues over time [18]. PFAS have been widely used in industrial and consumer applications, including non-stick cookware, food packaging, firefighting foams, and textile treatment, resulting in widespread environmental contamination and human exposure [17,19].

The endocrine-disrupting properties of PFASs have been increasingly recognized, with mounting evidence linking exposure to alterations in human reproductive hormone levels. Perfluorooctanoic acid (PFOA) and perfluorooctane sulfonate (PFOS), two of the most extensively studied PFAS compounds, have been associated with altered sex hormone levels, including changes in estradiol, FSH, LH, and SHBG, as well as disrupted menstrual characteristics in females [20]. The mechanism underlying PFAS-induced endocrine disruption includes interference with steroid hormone receptor activity and disruption of hormone-binding proteins [17].

PFAS bioaccumulation in reproductive tissue is particularly concerning as these compounds can concentrate in the ovaries, testes, and placenta, potentially exerting direct effects on reproductive organs. Animal studies have demonstrated that PFAS exposure can alter gonadotropin hormones, disrupt follicular development, and impair fertility outcomes [21].

The persistent nature of PFASs means that even past exposure can continue to influence hormone levels long after initial contact, making these compounds particularly problematic for reproductive health. The inclusion of PFASs in this mixture analysis is therefore justified by their near-universal detection in NHANES participants, their shared environmental exposure routes with other chemical classes, and their well-documented mechanisms of reproductive HPG axis disruption.

### 1.4. Toxic Metals

Heavy metals, including lead (Pb), cadmium (Cd), and mercury (Hg), represent another major class of environmental contaminants with well-documented endocrine-disrupting properties [22]. These metals are released into the environment through human activities, mining operations, and combustion processes, leading to widespread contamination of air, water, and food sources. Unlike organic pollutants, metals do not degrade over time and can bioaccumulate in human tissue, particularly in organs such as the kidney, liver, and reproductive tissues [23,24].

The reproductive toxicity of heavy metals has been extensively documented in both human and animal studies. Lead exposure has been associated with decreased sperm quality, altered hormone levels, and increased risk of infertility in males [25]. In females, lead exposure has been linked to irregular menstrual cycles, increased risk of pregnancy complications, and altered reproductive hormone profiles [26]. Cadmium, primarily derived from tobacco smoke and dietary sources, can accumulate in reproductive organs and has been associated with reduced fertility and altered hormone levels in both sexes [27].

Mercury exposure, primarily through consumption of contaminated fish and other seafood, has been linked to disrupted reproductive hormone levels and adverse reproductive outcomes [28]. The mechanisms of metal-induced endocrine disruption include interference with hormone-synthesizing enzymes, oxidative stress-mediated damage to reproductive tissues, and direct binding to hormone receptors. The ability of metals to cross the placental barrier and accumulate in fetal tissues makes prenatal exposure particularly concerning for reproductive development [28,29]. Toxic metals are co-analyzed alongside PFASs, phthalates, and VOCs because NHANES data confirm simultaneous population-level exposure to all four classes, and their shared reproductive endocrine targets necessitate a mixture-based analytical approach.

### 1.5. Phthalates

Phthalates are a group of synthetic chemicals widely used as plasticizers to increase the flexibility and durability of plastic products. They are also found in personal care products, fragrances, and medical devices. Due to their non-covalent binding to plastic matrices, phthalates can leach from products into the environment and human body, resulting in ubiquitous exposure [30,31]. Common phthalates include di(2-ethylhexyl) phthalate (DEHP), dibutyl phthalate (DBP), and diethyl phthalate (DEP) [30], all of which have been detected in human urine, serum, and tissues [32]. The anti-androgenic properties of phthalates have made them a particular focus of reproductive health research. Phthalate exposure has been consistently associated with decreased testosterone levels, altered sperm quality, and increased risk of male reproductive disorders [14,27]. The anti-androgenic effects of phthalates appear to result from interference with testosterone synthesis, particularly by inhibiting key enzymes in the steroidogenic pathway [27]. In females, phthalate exposure has been associated with altered menstrual cycle characteristics, early puberty, and disrupted ovarian function [33]. Prenatal phthalate exposure is of particular concern due to the critical role of androgens in male sexual differentiation [34]. Studies have demonstrated associations between maternal phthalate exposure and male genital development abnormalities, reduced anogenital distance, and altered reproductive hormone levels [35]. The ubiquitous nature of phthalate exposure and its short biological half-life [30] means that exposure is continuous and persists throughout critical developmental periods. In studying the chemical exposome, phthalates are included because of their documented ubiquity alongside PFASs, metals, and VOCs, and their established mechanisms of anti-androgenic endocrine disruption that target the same reproductive hormone endpoints.

### 1.6. Volatile Organic Compounds (VOCs)

VOCs are a diverse group of chemicals that readily evaporate at room temperature, resulting in widespread environmental presence and human exposure via inhalation [36,37]. Common VOCs include benzene, toluene, xylene, and various chlorinated compounds, which are found in industrial solvents, gasoline, paint, adhesives, and household products. Occupational exposure to VOCs is common in industries such as manufacturing, automotive, and chemical production [36]. Other environmental exposure occurs through contaminated air and water sources [38]. The reproductive toxicity of VOCs has been demonstrated in both occupational and environmental exposure studies. Benzene exposure has been associated with menstrual irregularities, reduced fertility, and altered hormone levels in females. Toluene and xylene exposure have been linked to disrupted reproductive hormone levels and adverse pregnancy outcomes [39]. The mechanism of VOC-induced endocrine disruption includes interference with hormone synthesis, oxidative stress, oxidative tissue damage, and disruption of hormone receptor signaling pathways. The volatile nature of these compounds means that exposure can occur rapidly and extensively, particularly in occupational settings or areas with poor ventilation. The ability of many VOCs to cross biological membranes easily facilitates the disruption of human reproductive organs, where they can exert a direct effect on hormone-producing cells. VOCs are co-analyzed with PFASs, metals, and phthalates because real-world inhalation and dietary exposure scenarios involve simultaneous exposure to all four classes, and emerging evidence implicates benzene and xylene metabolites in reproductive endocrine disruption.

### 1.7. Complex Mixture Effects and Methodological Considerations

The four chemical classes examined in this study—PFASs, toxic metals, phthalates, and VOCs—were selected for co-analysis on the basis of three converging justifications: (1) co-occurrence in shared environmental media and exposure routes (air, drinking water, food, consumer products), confirmed by near-universal detection of chemicals from all four classes in NHANES biomonitoring data; (2) convergent endocrine targets, as all four classes share documented mechanisms of HPG axis disruption, steroidogenic enzyme inhibition, and hormone receptor interference, making their combined effect on the same reproductive hormone endpoints biologically plausible and scientifically important; and (3) methodological necessity, as traditional single-pollutant analyses fall short in characterizing the joint effects or interactions among co-occurring chemicals. Human exposure to environmental chemicals rarely occurs in isolation; instead, individuals are simultaneously exposed to complex mixtures of chemicals from multiple sources. The combined effects of these complex mixtures may be greater than, less than, or different from the sum of individual chemical effects, depending on the nature of interactions between mixture components. Additive effects occur when the combined impact equals the sum of the individual effects, while synergistic effects result in responses that exceed the sum of the individual effects. Conversely, antagonistic effects occur when one chemical reduces the toxicity of another, resulting in less-than-additive responses. Traditional single-pollutant analytical approaches are inadequate for characterizing mixture effects, as they cannot capture the complex interactions that occur in real-world exposure scenarios.

Advanced statistical methods, such as Bayesian Kernel Machine Regression (BKMR), have been deployed to address these limitations by allowing for nonlinear dose–response relationships and interactions between mixture components [40]. BKMR is particularly well-suited for environmental mixture analysis because it does not require a priori specification of interaction terms and can identify important mixture components through variable selection procedures [40,41].

The reproductive endocrine system may be particularly vulnerable to mixture effects due to the complex hormonal interactions that regulate reproductive function. Chemicals that target different aspects of the HPG axis may interact to produce effects that are not predictable from individual chemical studies [42]. For example, chemicals that interfere with hormone synthesis may interact with those that disrupt hormone receptor function, producing amplified effects on reproductive outcomes.

### 1.8. Knowledge Gaps and Study Rationale

Despite growing awareness of environmental impacts on reproductive health, significant knowledge gaps remain regarding the combined effect of PFASs, toxic metals, phthalates, and VOCs on reproductive hormone levels. Most previous studies have focused on single chemicals or chemical classes, limiting our understanding of real-world mixture effects. Additionally, few studies have employed advanced statistical methods to characterize complex, potentially nonlinear relationships inherent in environmental mixtures. The identification of critical exposure windows, vulnerable populations, and key mixture components is essential for developing targeted public health interventions and regulatory policies. Understanding mixture effects is particularly important for reproductive health, given the critical role of hormones in fertility development and long-term health outcomes. The increasing prevalence of reproductive disorders, declining fertility rates, and earlier onset of puberty in many populations have raised concerns about the potential role of environmental chemical exposures. The primary objective of this study is to investigate the combined effects of PFASs, toxic metals, phthalates, and VOCs on reproductive hormone levels using data from a large, nationally representative survey, employing the BKMR methodology. We aim to characterize the joint effects of these chemical mixtures while accounting for nonlinear dose–response relationships and potential interactions among mixture components. This research will contribute to our understanding of the environmental impacts on reproductive health and inform evidence-based strategies to protect vulnerable populations.

## 2. Materials and Methods

### 2.1. Study Population

This cross-sectional analysis utilized data from the 2017–2018 cycle of the National Health and Nutrition Examination Survey (NHANES), a nationally representative multistage probability survey of the noninstitutionalized civilian population of the United States. NHANES combines standardized interviews, physical examinations, and laboratory testing to assess the health and nutritional status of the U.S. population, and its survey design enables generalization of findings to the broader American population.

The 2017–2018 NHANES cycle included 9254 participants who completed both the interview and examination components. Not all participants had measurements available for every exposure or hormone variable, reflecting the subsampling design of NHANES: PFAS measurements were available for 1929 participants, toxic metal measurements for 6884 (for lead) and 7513 (for cadmium and mercury), phthalate metabolite measurements for 2762, and VOC measurements for 2840 (for benzene), 2856 (for mp-xylene), 2866 (for o-xylene) and 2855 (for dichlorobenzene). Missing data within each analytical subsample were addressed using multiple imputation by chained equations (MICE), as described in Section 2.5.1, to maximize use of available information and reduce bias from incomplete observations.

All NHANES participants provided written informed consent prior to participation. Study protocols were approved by the National Center for Health Statistics Research Ethics Review Board. The use of the de-identified NHANES data for this secondary analysis was determined to be exempt from institutional review board review at our institution, as it does not constitute human subjects research under federal regulations.

### 2.2. Reproductive Hormone Measurements

Serum reproductive hormone concentrations were measured in participants from the National Health and Nutrition Examination Survey using standardized laboratory procedures. Hormones analyzed included estradiol, progesterone, follicle-stimulating hormone (FSH), luteinizing hormone (LH), sex-hormone-binding globulin (SHBG), and anti-Müllerian hormone (AMH). Blood samples were collected during the NHANES examination and processed according to standardized protocols before shipment to the Centers for Disease Control and Prevention laboratory for analysis.

Steroid hormones, including estradiol and progesterone, were quantified using isotope dilution liquid chromatography–tandem mass spectrometry (ID-LC-MS/MS) on a SCIEX API 6500 triple quadrupole mass spectrometer (SCIEX, Framingham, MA, USA) coupled to a Shimadzu high-performance liquid chromatography system (Shimadzu Corporation, Kyoto, Japan), with electrospray ionization. Analytes were identified by chromatographic retention time and specific mass-to-charge transitions via selected reaction monitoring, with isotopically labeled internal standards to ensure high analytical specificity and accuracy. In contrast, FSH, LH, SHBG, and AMH were measured using chemiluminescent immunoassays on a Roche/Hitachi cobas e 411 analyzer with Elecsys reagents (Roche Diagnostics, Indianapolis, IN, USA), which rely on antigen–antibody reactions followed by chemiluminescent detection using a photomultiplier tube after sequential incubation steps. The lower limits of detection (LLOD) for selected hormones in the NHANES 2017–2018 dataset were 1.72 pg/mL for estradiol, 0.86 ng/dL for progesterone, 0.30 mIU/mL for FSH, 0.10 mIU/mL for LH, 0.35 nmol/L for SHBG, and 0.03 ng/mL for AMH. For measurements falling below the LLOD, values were imputed using the standard NHANES substitution approach, in which the LLOD is divided by the square root of two (LLOD/√2) to minimize bias while maintaining the distribution of the data [43].

### 2.3. Environmental Chemical Exposure Assessment

#### 2.3.1. PFAS Measurements

This study evaluated serum concentrations of perfluorooctanoic acid (PFOA) and perfluorooctane sulfonate (PFOS) measured in participants from the National Health and Nutrition Examination Survey. NHANES measured both linear PFOA (n-PFOA) and branched PFOA isomers (Sb-PFOA), as well as linear PFOS (n-PFOS) and monomethyl-branched PFOS isomers (Sm-PFOS). For this analysis, the linear and branched isomers of PFOA were summed to yield a single total PFOA variable, and similarly, the isomers of PFOS were summed to yield a single total PFOS variable. Analyses and tables, therefore, present PFOA and PFOS as single summed variables rather than individual isomers. Serum samples were processed using solid-phase extraction followed by high-performance liquid chromatography and tandem mass spectrometry (SPE-HPLC-MS/MS) with negative-ion turbo ion spray ionization on a SCIEX mass spectrometer (SCIEX, Framingham, MA, USA), providing high specificity and sensitivity for low-nanogram-per-milliliter concentrations. Approximately 50 μL of serum, diluted with formic acid, was introduced into a column-switching system to concentrate analytes prior to chromatographic separation. Values below the lower limit of detection (0.10 ng/mL) were imputed using the standard substitution method (LOD/√2) to maintain statistical validity [44].

#### 2.3.2. Toxic Metal Analysis

Blood concentrations of heavy metals were measured in participants from the National Health and Nutrition Examination Survey. The analytes included lead (Pb), cadmium (Cd), and mercury (Hg). Whole-blood samples were collected in EDTA tubes (Becton, Dickinson and Company, Franklin Lakes, NJ, USA) to prevent coagulation and ensure uniform distribution of blood components. Analysis was performed using inductively coupled plasma–dynamic reaction cell–mass spectrometry (ICP-DRC-MS) on a PerkinElmer ELAN DRC II instrument (PerkinElmer, Inc., Shelton, CT, USA). Samples were diluted with a reagent mixture that solubilizes blood components and releases metals. A high-temperature argon plasma ionized metal atoms, which then passed through a dynamic reaction cell to reduce interferences before detection by a quadrupole mass filter and electron multiplier detector. Rhodium, tellurium, and iridium were used as internal standards to correct for matrix effects and instrument drift. Concentrations were determined using calibration curves following NHANES quality control protocols to ensure accuracy and reproducibility. Measurements below the limits of detection (LOD) were imputed using the standard substitution method, where LOD values are divided by the square root of 2 (LOD/√2). Detection limits for this analysis were 0.07 μg/dL for Pb, 0.10 μg/L for Cd, and 0.28 μg/L for total Hg [44].

#### 2.3.3. Phthalates Metabolite Measurements

Urinary phthalate metabolites were measured in participants from the National Health and Nutrition Examination Survey to assess exposure to commonly used phthalate plasticizers. Urine samples were analyzed for multiple metabolites derived from widely used parent compounds, including di-(2-ethylhexyl) phthalate (DEHP), dibutyl phthalate (DBP), and diethyl phthalate (DEP). Quantification was performed using high-performance liquid chromatography coupled with electrospray ionization tandem mass spectrometry (HPLC-ESI-MS/MS) following enzymatic deconjugation and online solid-phase extraction. This procedure releases glucuronidated metabolites and concentrates analytes prior to chromatographic separation. Detection was conducted using multiple reaction monitoring with isotopically labeled internal standards to ensure analytical accuracy and precision. The method provides high sensitivity suitable for biomonitoring of environmental exposures, with detection limits generally in the low nanogram per milliliter (ng/mL) range, depending on the metabolite.

When measured concentrations fell below the limit of detection (LOD), imputed values were calculated using the standard substitution approach applied in NHANES biomonitoring datasets, where values are replaced by the LOD divided by the square root of two (LOD/√2) to minimize bias in statistical analyses [45].

#### 2.3.4. VOC Measurement

Blood concentrations of volatile organic compounds (VOCs) were measured in participants from the National Health and Nutrition Examination Survey to evaluate exposure to common environmental and industrial chemicals. Whole-blood specimens were analyzed for a broad panel of VOCs, including compounds such as Benzene, Toluene, Ethylbenzene, and Xylene isomers, as well as chlorinated solvents and other hydrocarbons frequently encountered in environmental exposures. Analysis was performed using headspace solid-phase microextraction (SPME) followed by capillary gas chromatography coupled with isotope-dilution mass spectrometry (GC-MS). In this procedure, a portion of whole blood is transferred into a sealed headspace vial, allowing VOCs to partition into the headspace above the sample [46]. A coated SPME fiber is then exposed to the headspace to adsorb the volatile compounds, after which the fiber is introduced into the heated gas chromatograph inlet where the analytes are thermally desorbed and separated on a capillary column. Detection and quantification are performed using mass spectrometry with selected-ion monitoring and isotopically labeled internal standards to ensure high analytical specificity and accuracy. This analytical method provides high sensitivity suitable for biomonitoring studies, with detection limits in the low parts-per-trillion range in whole blood, enabling quantification of VOC concentrations typically observed in the general population. Rigorous quality control procedures and calibration with isotopically labeled standards were used to ensure the reliability and reproducibility of measurements. For analyte concentrations below the limit of detection (LOD), values were imputed using the standard substitution approach applied in NHANES biomonitoring datasets, where the LOD is divided by the square root of two to reduce bias in statistical analyses [47].

### 2.4. Covariate Assessment

Demographic, lifestyle, and health information were collected through standardized questionnaires administered by trained interviewers. Covariates included in the analysis were selected based on their potential to confound relationships between environmental exposures and reproductive hormone levels. These included age, sex, race/ethnicity, body mass index (BMI), smoking status, alcohol consumption, diabetes, annual income, hypertension, and steroid use. Age was analyzed as a continuous variable, sex as male or female, and race/ethnicity as NHANES categories: Non-Hispanic white, Non-Hispanic Black, Mexican–American, Other Hispanic, Non-Hispanic Asian, and Other/Multiracial. Smoking status was categorized as current smoker (Yes) or non-smoker (No) [48]. These factors can influence hormone levels and were included to reduce residual confounding in the analysis. Missing covariate data were addressed through multiple imputation procedures as described below.

### 2.5. Statistical Analysis

#### 2.5.1. Multiple Imputation for Missing Data

Missing data for exposure variables and covariates were addressed using multiple imputation by chained equations (MICE) in R with the mice package. This approach assumes that data are missing at random (MAR) conditional on observed variables and generates multiple complete datasets through iterative imputation procedures. It is important to acknowledge that missingness in NHANES arises from two structurally distinct mechanisms. The dominant source is design-driven subsampling, in which specific biomarker panels are measured only in pre-specified probability subsamples selected based on fully observed demographic and logistical criteria. Conditional on the observed design variables, age, sex, race/ethnicity, and examination half-sample, all of which were included as auxiliary variables in the imputation model, this component of missingness is missing at random (MAR) by construction. A secondary, smaller source of missingness arises from item nonresponse, which may in some instances be missing not at random (MNAR) if, for example, health status or exposure burden influences willingness to provide biological specimens. While we cannot formally rule out MNAR for this component, MAR-based MICE remains the most defensible approach given that the following considerations: (1) design-driven subsampling accounts for the large majority of missing values in this dataset (e.g., PFAS subsample *n* = 1929 vs. total *n* = 9254); (2) all variables known to predict missingness—including age, sex, race/ethnicity, BMI, income, and NHANES examination half-sample—were included as imputation model auxiliaries, minimizing residual MNAR risk; (3) formal MNAR sensitivity analyses (e.g., pattern mixture models or selection models) require untestable parametric assumptions and are computationally prohibitive given the complexity of the 28-variable exposure dataset; and (4) a complete-case sensitivity analysis produced results directionally consistent with the imputed estimates, suggesting any MNAR bias is unlikely to materially alter the substantive conclusions. The potential for residual MNAR bias is acknowledged as a limitation of this study. The imputation model included all variables to be used in subsequent analysis as well as auxiliary variables that were correlated with missingness pattern or outcome variables. Separate imputation models were developed for continuous and categorical variables with appropriate distribution assumptions for continuous variables. Predictive mean matching was used while logistic or multinomial regression was employed for binary and categorical variables, respectively. Twenty imputed datasets were generated with convergence of the imputation algorithms assessed through trace plots and other diagnostic procedures. Analyses were performed on each imputed dataset separately, with results combined using Rubin’s rules to account for imputation uncertainty in standard error calculation and significance testing [49,50].

##### General Framework

MICE imputes missing data in a variable-by-variable manner, using a sequence of regression models.

For a dataset $\text{X}=\left({\text{X}}_{1},{\text{X}}_{2},\ldots ,{\text{X}}_{p}\right)$, where some variables have missing values:

At iteration t:

$${X_{j}}^{\left(t\right)}\sim f_{j}\left(X_{j}|X_{-j}\right)$$

where

${\text{X}}_{\text{j}}$ = variable being imputed;

${\text{X}}_{-\text{j}}$ = all other variables except Xj;

${\text{f}}_{\text{j}}(.)$ = conditional distribution for variable X given the others.

This process cycles through each variable with missing data, updating imputations iteratively until convergence.

##### Equation by Variable Type

- Continuous variable—linear regression imputation.
  For variable ${\text{X}}_{\text{j}}$:
$$X_{j}=\beta_{0}+\sum \beta_{k}X_{k}+\varepsilon ,\;\varepsilon \sim N\left(0,\sigma^{2}\right)$$
  Imputed value:
$${X_{j}}^{\left(t\right)}\sim N\left({\overset{ˆ}{Y}}_{j}^{\left(t\right)},\sigma^{2}\right)$$
  where $\overset{ˆ}{Y}\text{j}(\text{t})=\beta 0+\sum (\beta \text{k}\;\text{Xk})$ is the predicted mean from the regression model at iteration t.
- Binary variable—logistic regression imputation.
  For binary ${\text{X}}_{\text{j}}\in$:
$$\text{logit}\left(P\left(X_{j}=1\right)\right)=\beta_{0}+\sum \beta_{k}X_{k}$$
  Imputed values
$$\text{X}ˇ{\text{c}}^{\left(\text{t}\right)}\sim \text{Bernoulli}\left(\text{p}ˇ{\text{c}}^{\left(\text{t}\right)}\right),\;\text{where}\;\text{p}ˇ{\text{c}}^{\left(\text{t}\right)}=1/\left(1+{\text{e}}^{-}\left(\beta_{0}+\sum \beta_{\text{k}}\;{\text{X}}_{\text{k}}\right)\right)$$
- Categorical variable (multinomial logistic regression).
  For ${\text{X}}_{\text{j}}$ with K categories:
$$P\left(X_{j}=k\right)=\frac{\text{exp}\left(\beta_{0k}+\sum \beta_{\text{lk}}X_{l}\right)}{\sum_{\text{x}}\;\text{exp}\left(\beta_{0m}+\sum \beta_{\text{lm}}X_{l}\right)}$$
  Impute ${{\text{X}}_{\text{j}}}^{\left(\text{t}\right)}$ by sampling from this multinomial distribution.
  Then, sample imputed values based on cumulative probabilities.

#### 2.5.2. Bayesian Kernel Machine Regression (BKMR)

The primary analytical approach employed BKMR to evaluate the joint effects of environmental chemical mixtures on reproductive hormone levels. BKMR is a flexible statistical method that accommodates nonlinear exposure–response relationships and interactions among mixture components without requiring a priori specification of the functional form.

The BKMR model can be expressed as

$$Y_{i}=h\left(z_{i}\right)+{X_{i}}^{T}\beta +\varepsilon_{i}$$

where $Y_{i}$ represents the reproductive hormone concentration for individual $i,z_{i}$ is a vector of environmental exposures, and $X_{i}$ is a vector of covariates. The unknown exposure–response function h(·) is flexibly modeled using a Bayesian Gaussian process kernel function, allowing for nonlinear and interactive effects among mixture components. The residual errors are assumed to follow a normal distribution, $\varepsilon_{i}\sim N\left(0,\sigma^{2}\right)$.

The Kernel machine approach utilizes a Gaussian kernel to capture complex relationships between exposure and outcomes. Bayesian variable selection procedures identify important mixture components through posterior inclusion probabilities (PIP), which quantify the probability that each exposure contributes to the outcome. Markov chain Monte Carlo (MCMC) sampling was used for posterior inference, with model convergence assessed through a standard diagnostic procedure.

BKMR analysis provides several types of output relevant to mixture analysis: (i) univariate exposure–response functions showing the relationship between individual exposure and outcome while holding other exposures at their median value; (ii) single-variable summary plots comparing effects at the 75th versus 25th percentile of each exposure; (iii) single-exposure-attributable effects across background mixture quantiles.

Overall mixture effects comparing outcomes at different quantiles of the overall mixture distribution and individual variable importance measures through PIPs.

Model fitting used 10,000 MCMC iterations, with a 5000-iteration burn-in. Convergence was assessed using trace plots of model parameters across all six hormone-specific models; all chains demonstrated stable mixing and no systematic trends after the burn-in period, indicating satisfactory convergence. Posterior distributions were summarized using posterior means and 95% credible intervals. All analyses were adjusted for relevant covariates selected based on prior knowledge of confounding relationships and established epidemiological evidence.

All models included sex as a covariate to account for fundamental differences in reproductive hormone physiology between males and females. Sex was retained as a covariate rather than a stratification variable for two primary reasons: (1) BKMR with 28 predictors requires an adequate sample size for stable posterior estimation, and stratification would halve the effective sample size per stratum; and (2) sex as a covariate captures the primary between-sex difference in hormone levels while allowing characterization of population-average mixture associations. Sex-stratified BKMR analyses are recommended as a priority for future work. Results are presented for the full analytical sample, with sex-specific differences captured through covariate adjustment rather than formal stratification, given the broad age range and the availability of hormone measurements across both sexes in the NHANES dataset.

For multivariable linear regression analyses, NHANES survey weights, strata, and primary sampling units were incorporated using the survey package in R to account for the complex multistage sampling design. Because the bkmr package does not natively support complex survey weights, BKMR models were fit in the analytical sample without survey weights; therefore, BKMR results should be interpreted as sample-based mixture associations rather than fully survey-weighted population estimates. Exposure variables were analyzed in their original NHANES measurement units for multivariable linear regression. Regression coefficients, therefore, represent changes in hormone concentrations per one-unit increase in each exposure biomarker and should be interpreted in relation to the exposure-specific interquartile range, particularly for highly skewed VOC biomarkers. For BKMR analyses, exposure variables were standardized prior to model fitting to place chemicals measured on different scales onto a comparable metric for kernel estimation. No log transformation was applied unless otherwise stated.

Statistical analyses were performed using R version 4.3.0 with the bkmr package used for BKMR implementations. Graphics were generated using ggplot2 and other visualization packages, with results presented according to established guidelines for environmental measures and analysis.

## 3. Results

### 3.1. Summary of Key Study Variables

In this study, we analyzed reproductive hormone profiles and environmental chemical exposures among 9254 participants from the NHANES dataset. Hormone concentrations were reported in the following units: estradiol in pg/mL, FSH and LH in mIU/mL, progesterone in ng/dL, SHBG in nmol/L, and AMH in ng/mL, consistent with standard NHANES laboratory reporting. Environmental exposure concentrations were reported as follows: serum PFAS (PFOA, PFOS) in ng/mL; whole-blood lead in μg/dL; whole-blood cadmium and mercury in μg/L; urinary phthalate metabolites in ng/mL; and whole-blood VOC metabolites (benzene, mp-xylene, o-xylene, dichlorobenzene) in ng/mL. These units are used consistently throughout all tables and figures. The cohort demonstrated substantial demographic heterogeneity among participants (mean age: 34.3 years, SD: 25.5). Body mass index measurements averaged 26.6 kg/m^2^ (SD: 8.3), representing diverse body compositions across the study sample.

Table 1 summarizes the distribution of environmental exposures and demographic characteristics across the study population. Among PFASs compounds, PFOS concentrations (mean: 6.5 ng/mL, SD: 7.7) substantially exceeded PFOA levels (mean: 1.7 ng/mL, SD: 1.8), reflecting historical patterns of industrial production and environmental persistence. Regarding toxic metal exposures, mercury exhibited the highest mean concentration (1.14 μg/L, SD: 2.27), while lead (mean: 1.08 μg/dL, SD: 1.29) and cadmium (mean: 0.37 μg/L, SD: 0.50) demonstrated lower but consistently detectable levels throughout the population.

Phthalate metabolite concentrations revealed the broadest exposure spectrum, with MEP displaying pronounced variability (mean: 149 ng/mL, SD: 2016), indicative of widespread exposure through personal care products and consumer goods. High-molecular-weight phthalate metabolites, including MEHHP and MEHP, registered at moderate concentrations, signaling continued exposure to plasticizers prevalent in food packaging and household products. Volatile organic compound concentrations remained comparatively low, though inter-individual variation suggested diverse exposure sources across the population.

Figures 1 and 2 present complementary correlation heatmaps examining pairwise relationships among environmental chemical exposures and reproductive hormone biomarkers. Figure 1 uses Spearman rank-based correlations, which are more robust to outliers and non-normal distributions, both common features of environmental biomarker data, while Figure 2 uses Pearson correlations to characterize strictly linear relationships. Presenting both allows identification of associations that are monotonic but nonlinear (detected by Spearman but not Pearson) versus those that are robustly linear (detected by both), providing a more complete characterization of the exposure–hormone correlation structure. The strength of correlations is represented visually, with darker red tones indicating strong positive associations and darker blue tones representing strong negative associations. Following established guidelines, correlations were classified as weak (|r| < 0.3), moderate (0.3 ≤ |r| < 0.5), and strong (|r| ≥ 0.5).

Within phthalate metabolite groups, strong positive correlations (r = 0.4–0.7) emerged among DEHP metabolites (MEHP, MEOHP, MEHHP), reflecting their shared origin from di(2-ethylhexyl) phthalate metabolism. This clustering pattern aligns with common exposure pathways involving dietary intake and consumer product use. PFAS compounds demonstrated moderate intercorrelation (r ≈ 0.5 between PFOS and PFOA), suggesting co-occurrence in contaminated water supplies and consumer products.

Toxic metals exhibited weaker yet meaningful associations, with lead and cadmium showing a modest positive correlation (r ≈ 0.19), potentially indicating shared environmental sources such as industrial emissions or tobacco smoke exposure. However, cross-class correlations among PFASs, metals, phthalates, and VOCs remained minimal (r < 0.2), suggesting these chemical categories represent largely independent exposure domains within the overall mixture environment. Importantly, cross-chemical-class correlations were uniformly weak (r < 0.20 for all PFAS–metal, PFAS–phthalate, PFAS–VOC, metal–phthalate, metal–VOC, and phthalate–VOC pairs), providing formal empirical support for treating the four chemical classes as largely independent exposure domains within the BKMR mixture model. The strong within-class clustering observed for DEHP metabolites and PFAS compounds reflects shared metabolic pathways; this collinearity means that posterior inclusion probabilities for correlated chemicals within the same class should be interpreted collectively rather than in isolation.

Regarding hormone associations, estradiol demonstrated predominantly weak or inverse correlations with most environmental exposures. PFAS compounds showed negative relationships with estradiol, supporting experimental evidence of anti-estrogenic mechanisms. Phthalate metabolites and VOCs exhibited near-zero or marginally positive associations, while metal exposures showed negligible linear relationships. Sex-hormone-binding globulin displayed moderate positive correlations with several PFAS compounds and select phthalate metabolites. These patterns suggest complex, potentially nonlinear mechanisms underlying reproductive hormone disruption that necessitate advanced mixture modeling approaches.

Figure 2 presents a Pearson correlation heatmap showing pairwise linear relationships among environmental chemicals and reproductive hormone biomarkers. Darker red tones indicate stronger positive correlations, while darker blue tones reflect stronger negative correlations. Correlations were classified as weak (|r| < 0.3), moderate (0.3 ≤ |r| < 0.5), and strong (|r| ≥ 0.5).

The heatmap shows strong positive correlations among several phthalate metabolites, indicating shared sources or metabolic pathways. VOCs such as benzene and the xylenes demonstrated moderate correlations with each other but weak correlations with most other chemicals. Metals (lead, cadmium, and mercury) showed generally weak associations across the matrix. Hormone–chemical correlations were mostly weak, with a moderate positive association observed between estradiol and progesterone. Overall, the results showed distinct clustering within chemical groups but minimal linear relationships between individual chemicals and hormone biomarkers.

### 3.2. Regression Results for Key Reproductive Hormones

Table 2 presents the results from multivariable linear regression analyses examining associations between individual environmental chemicals and reproductive hormone concentrations. All models were adjusted for covariates as described in Section 2.4.

#### 3.2.1. Estradiol

For estradiol, numerous environmental chemicals demonstrated statistically significant associations across multiple chemical classes. Among metals, lead exhibited a positive association (β = 2.54; 95% CI: 1.11, 3.98), while mercury showed a significant inverse relationship (β = −3.38; 95% CI: −5.12, −1.65). Among phthalate metabolites, positive associations were observed for MCNP (β = 1.30; 95% CI: 0.61, 2.00), MBP (β = 0.83; 95% CI: 0.32, 1.34), MCOCH (β = 6.86; 95% CI: 5.70, 8.02), MEHHP (β = 1.79; 95% CI: 1.00, 2.58), and MHHT (β = 0.44; 95% CI: 0.12, 0.76), whereas MCPP (β = −0.81; 95% CI: −0.98, −0.65), MHNCH (β = −8.44; 95% CI: −9.82, −7.07), MEHP (β = −6.46; 95% CI: −10.34, −2.59), and MHBP (β = −8.72; 95% CI: −16.03, −1.41) exhibited significant inverse associations, and MONP (β = −1.02; 95% CI: −2.00, −0.03) exhibited a nominally significant inverse association that did not survive FDR correction. Volatile organic compounds also revealed notable relationships: benzene showed a nominally significant positive association (β = 62.0; 95% CI: 1.88, 122.13) that did not survive FDR correction, and dichlorobenzene showed a positive association (β = 0.59; 95% CI: 0.22, 0.95), while mp-xylene demonstrated a nominally significant inverse association (β = −69.92; 95% CI: −128.21, −11.63) that did not survive FDR correction. These findings indicate heterogeneous effects across and within chemical classes, with certain exposures potentially disrupting estrogen synthesis or metabolism while others may upregulate estradiol synthesis or inhibit its hepatic metabolism.

#### 3.2.2. Luteinizing Hormone

Luteinizing hormone demonstrated numerous associations across all four chemical classes, several of which were statistically significant after FDR correction, and others that were only nominally significant. Among PFASs, PFOA displayed a nominally significant positive association (β = 6.91; 95% CI: 1.44, 12.38) that did not survive FDR correction. Among metals, cadmium showed a nominally significant positive association with LH (β = 16.18; 95% CI: 1.84, 30.52) that did not survive FDR correction. Among phthalate metabolites, significant positive associations were observed for MECP (β = 2.45; 95% CI: 1.56, 3.34), MHIBP (β = 12.95; 95% CI: 4.17, 21.73), MHNCH (β = 5.18; 95% CI: 2.39, 7.96), and MONP (β = 2.33; 95% CI: 0.54, 4.12), while MEHP showed a nominally significant positive association (β = 7.91; 95% CI: 0.90, 14.91) that did not survive FDR correction. MCNP (β = −1.41; 95% CI: −2.55, −0.27), MCPP (β = −0.32; 95% CI: −0.63, −0.003), and MiBP (β = −1.97; 95% CI: −3.76, −0.18) showed nominally significant inverse associations that did not survive FDR correction, while MCOCH (β = −5.26; 95% CI: −7.51, −3.01) and MNP (β = −17.87; 95% CI: −27.66, −8.08) showed significant inverse associations. Among VOCs, mp-xylene showed a nominally significant positive relationship (β = 156.42; 95% CI: 29.97, 282.87) with LH concentrations that did not survive FDR correction, while o-xylene showed a nominally significant inverse relationship with LH (β = −423.44; 95% CI: −778.76, −68.12) that did not survive FDR correction, and dichlorobenzene also showed a significant inverse association (β = −0.79; 95% CI: −1.43, −0.15). These divergent patterns across and within chemical classes highlight the complexity of endocrine disruption mechanisms affecting gonadotropin regulation. Note: the large coefficient magnitudes observed for VOC exposures (particularly mp-xylene and o-xylene) reflect the raw blood concentration scale (ng/mL) and the wide distributional range of these biomarkers; when interpreted relative to the interquartile range of each VOC, the effect sizes are considerably more modest.

#### 3.2.3. Sex-Hormone-Binding Globulin

Sex-hormone-binding globulin exhibited the most extensive pattern of significant individual chemical associations among all hormones examined. Among PFASs, both PFOA (β = 12.35; 95% CI: 8.33, 16.38) and PFOS (β = 1.03; 95% CI: 0.12, 1.94) showed significant positive associations. Among metals, lead showed a significant inverse association (β = −5.23; 95% CI: −7.64, −2.82). Among phthalate metabolites, significant positive associations were observed for MCNP (β = 2.57; 95% CI: 1.51, 3.62), MECP (β = 1.42; 95% CI: 0.65, 2.19), MHHT (β = 2.49; 95% CI: 2.01, 2.96), MHNCH (β = 7.04; 95% CI: 5.12, 8.96), MEHP (β = 9.44; 95% CI: 3.08, 15.80), MEOHP (β = 15.47; 95% CI: 11.78, 19.15), MONP (β = 4.72; 95% CI: 3.16, 6.28), and MBzP (β = 1.07; 95% CI: 0.59, 1.55), while MCOP (β = −0.93; 95% CI: −1.24, −0.62), ECPT (β = −0.41; 95% CI: −0.49, −0.33), MCPP (β = −1.36; 95% CI: −1.64, −1.08), MCOCH (β = −6.83; 95% CI: −8.24, −5.42), MiBP (β = −2.52; 95% CI: −4.04, −1.00), and MNP (β = −17.45; 95% CI: −25.37, −9.53) showed significant inverse associations. Among VOCs, mp-xylene showed a striking positive association (β = 524.75; 95% CI: 420.02, 629.47) and dichlorobenzene also showed a positive association (β = 0.71; 95% CI: 0.19, 1.22), while o-xylene showed a significant inverse association (β = −1411.40; 95% CI: −1708.59, −1114.21). The predominance of positive associations across chemical classes suggests many environmental chemicals may induce compensatory upregulation of SHBG, potentially reflecting hepatic responses to endocrine disruption or alterations in hormone transport protein synthesis. The large regression coefficients observed for VOC exposures (particularly mp-xylene and o-xylene with LH and SHBG) reflect the raw blood concentration measurement scale (ng/mL). These should be interpreted in the context of each VOC’s interquartile range, within which the actual population-level effect magnitudes are considerably more modest. The BKMR exposure–response curves corroborate that VOC effects at typical population exposure levels are modest.

#### 3.2.4. Follicle-Stimulating Hormone

Follicle-stimulating hormone demonstrated numerous statistically significant associations across multiple chemical classes. Among phthalate metabolites, significant positive associations were observed for MECP (β = 4.09; 95% CI: 2.47, 5.72), MHIBP (β = 22.15; 95% CI: 7.19, 37.12), and MHNCH (β = 12.52; 95% CI: 7.38, 17.65); MEHP showed a nominally significant positive association (β = 16.60; 95% CI: 2.86, 30.34) that did not survive FDR correction. MCOCH (β = −11.45; 95% CI: −15.37, −7.53), MEHHP (β = −11.26; 95% CI: −14.04, −8.48), and MNP (β = −20.71; 95% CI: −35.37, −6.05) showed significant inverse associations, while MCNP (β = −2.32; 95% CI: −4.40, −0.25) and MHHT (β = −1.28; 95% CI: −2.30, −0.25) showed nominally significant inverse associations that did not survive FDR correction. Among VOCs, dichlorobenzene demonstrated a nominally significant inverse association (β = −1.18; 95% CI: −2.23, −0.13) that did not survive FDR correction. PFOA showed a positive but non-significant association with FSH (β = 8.45; 95% CI: −1.25, 18.14), though it was identified as a key PFAS predictor in the BKMR PIP analysis. Neither mercury nor cadmium showed statistically significant associations with FSH (mercury: β = −0.72; 95% CI: −6.65, 5.22; cadmium: β = 26.26; 95% CI: −0.40, 52.93), consistent with the absence of significant metal effects on FSH in the linear regression framework.

#### 3.2.5. Anti-Müllerian Hormone

Anti-Müllerian hormone showed numerous associations across all four chemical classes, most of which were statistically significant after FDR correction. Among PFASs, PFOA showed a significant inverse association (β = −1.80; 95% CI: −2.43, −1.17), while PFOS showed a significant positive association (β = 0.18; 95% CI: 0.05, 0.30). Among metals, cadmium (β = −3.02; 95% CI: −4.73, −1.31) and lead (β = −0.90; 95% CI: −1.60, −0.21) showed significant inverse associations with AMH, while mercury demonstrated a significant positive association (β = 0.64; 95% CI: 0.15, 1.12). Among phthalate metabolites, significant positive associations were observed for MCNP (β = 0.31; 95% CI: 0.09, 0.52), MCPP (β = 0.04; 95% CI: 0.01, 0.08), MEHHP (β = 0.64; 95% CI: 0.31, 0.97), MHHT (β = 0.12; 95% CI: 0.03, 0.22), MHBP (β = 2.21; 95% CI: 0.18, 4.23), and MiBP (β = 0.42; 95% CI: 0.16, 0.68), while MHIBP showed a nominally significant inverse association (β = −2.59; 95% CI: −3.60, −1.58) that did not survive FDR correction. MEHP did not show a statistically significant association with AMH (β = −1.21; 95% CI: −2.52 to 0.09). Among VOCs, benzene showed a significant positive association (β = 37.04; 95% CI: 20.53, 53.55). These heterogeneous patterns across and within chemical classes are consistent with diverse mechanisms of EDC action on ovarian granulosa cell function and AMH synthesis, with the contrasting directions of PFOA (inverse) and PFOS (positive) being particularly noteworthy given their structural similarity.

#### 3.2.6. Progesterone

Progesterone exhibited the richest pattern of individual chemical associations among all hormones examined. Multiple significant inverse associations were observed, including PFOA (β = −6.30; 95% CI: −7.60, −4.99), cadmium (β = −17.73; 95% CI: −22.48, −12.98), lead (β = −2.24; 95% CI: −3.02, −1.47), MHIBP (β = −5.21; 95% CI: −7.91, −2.51), MEHHP (β = −4.55; 95% CI: −6.74, −2.36), MEHP (β = −1.23; 95% CI: −1.62, −0.83), MCOP (β = −0.27; 95% CI: −0.38, −0.15), ECPT (β = −0.07; 95% CI: −0.10, −0.05), and MCOCH (β = −0.72; 95% CI: −1.39, −0.05), the latter a nominally significant association that did not survive FDR correction, suggesting potential suppressive effects on corpus luteum function or steroidogenic enzyme activity. Conversely, significant positive associations included PFOS (β = 0.84; 95% CI: 0.54, 1.15), mercury (β = 0.99; 95% CI: 0.14, 1.85), MCNP (β = 1.18; 95% CI: 0.78, 1.57), MECP (β = 0.37; 95% CI: 0.11, 0.63), MHHT (β = 0.75; 95% CI: 0.57, 0.92), MEOHP (β = 2.40; 95% CI: 1.46, 3.34), MONP (β = 0.71; 95% CI: 0.18, 1.23), MBzP (β = 0.37; 95% CI: 0.21, 0.52), and benzene (β = 97.58; 95% CI: 63.59, 131.57). These contrasting within-class and cross-class patterns underscore the complexity of EDC mechanisms affecting reproductive steroid synthesis and are consistent with the pronounced emergent mixture effects subsequently identified in the BKMR overall mixture analysis.

### 3.3. Bayesian Kernel Machine Regression Results

Within the Bayesian Kernel Machine Regression framework, this study evaluated PIPs, univariate exposure–response relationships, overall mixture effects, and single-variable attributable effects. Outcome variables comprised reproductive hormone biomarkers, while predictor variables included environmental chemicals across multiple classes. Covariates, including age, sex, race/ethnicity, body mass index, smoking status, alcohol consumption, diabetes, annual income, hypertension, and steroid use, were incorporated into all analyses. Results of these analyses are detailed in subsequent sections.

#### 3.3.1. Posterior Inclusion Probability

Table 3 summarizes PIPs from the hierarchical BKMR model. The environmental chemicals were organized into four pre-specified exposure groups: Group 1 = PFAS (PFOA, PFOS); Group 2 = Toxic Metals (lead, cadmium, mercury); Group 3 = Phthalate metabolites (19 urinary metabolites); and Group 4 = VOC metabolites (benzene, mp-xylene, o-xylene, and dichlorobenzene). Two PIP metrics are reported. The group PIP reflects the probability that at least one chemical in the group is an important predictor of the hormone outcome, capturing class-level relevance regardless of which specific chemical drives the effect. The conditional PIP reflects the probability that a specific chemical is an important predictor given that its group has already been selected, identifying the dominant individual driver within each class. Conditional PIPs should be interpreted in the context of their corresponding group posterior inclusion probability. A high conditional PIP carries limited inferential weight when its group PIP is low, since it is conditioned on an event of low probability.

At the group level, phthalate metabolites (Group 3) showed the most consistent class-level relevance, with group PIPs of 1.0 across all six hormone endpoints, indicating near-certain importance of this chemical class for every reproductive hormone examined. Metals (Group 2) showed uniformly high group PIPs for estradiol, FSH, progesterone, and AMH (all 1.0), with moderate values for LH (0.876) and SHBG (0.560). PFASs (Group 1) showed high group PIPs for estradiol, FSH, progesterone, and AMH (all 1.0), a moderate value for SHBG (0.607), and a markedly low group PIP for LH (0.07), indicating that the PFAS class had negligible collective relevance for LH regulation; conditional PIPs within the PFAS group for LH are therefore not interpretable. VOCs (Group 4) showed high group PIPs for estradiol, LH, and AMH (all 1.0) and a moderate value for progesterone (0.902), but uncertain class-level relevance for FSH (0.640) and SHBG (0.504).

At the level of individual chemicals, conditional PIPs revealed highly selective patterns both within and across groups. Within the PFAS group, PFOA was the dominant predictor for FSH (condPIP = 1.0), progesterone (1.0), SHBG (1.0), and AMH (1.0). For estradiol, however, conditional importance within the PFAS group shifted entirely to PFOS (condPIP = 1.0), with PFOA contributing negligibly (condPIP = 0.0). Within the metals group, each metal contributed selectively to distinct hormone endpoints. Cadmium was the primary metal driver for estradiol (condPIP = 1.0) and LH (0.977). Mercury was the dominant metal predictor for progesterone (condPIP = 1.0), though its conditional importance for SHBG (condPIP = 1.0) should be interpreted with caution, given the moderate metals group PIP for that endpoint (0.560). Lead dominated within the metals group for FSH (condPIP = 0.996) and AMH (1.0). Within the phthalate group, despite uniformly high group PIPs of 1.0 across all hormones, conditional importance concentrated entirely in three of the 19 metabolites; the remaining 16 had conditional PIPs of 0.0 across all endpoints. MBzP achieved condPIP = 1.0 for estradiol, LH, and AMH. MHIBP was the sole phthalate driver for progesterone (condPIP = 1.0), and MEHP was the primary phthalate predictor for SHBG (condPIP = 1.0). Within the VOC group, mp-xylene was the dominant predictor for estradiol (condPIP = 0.909) and the sole VOC driver for LH (condPIP = 1.0). Benzene was the principal VOC predictor for AMH (condPIP = 0.9996)—the strongest VOC conditional finding in the entire table, and for SHBG (condPIP = 0.933), though the moderate VOC group PIP for SHBG (0.504) warrants caution in interpreting the latter. Dichlorobenzene showed the highest conditional importance within the VOC group for progesterone (condPIP = 0.720), supported by a high VOC group PIP for that endpoint (0.902). For FSH, VOC conditional PIPs were distributed across o-xylene (0.521), dichlorobenzene (0.270), and mp-xylene (0.168), with no single VOC emerging as a clear driver, and the uncertainty is compounded by the moderate VOC group PIP for FSH (0.640).

#### 3.3.2. Univariate, Overall, Single-Variable, and Interactive Effects

We used BKMR analyses to examine associations between environmental chemical mixtures and all six reproductive hormone biomarkers: estradiol, luteinizing hormone (LH), follicle-stimulating hormone (FSH), sex-hormone-binding globulin (SHBG), anti-Müllerian hormone (AMH), and progesterone. For each hormone endpoint, a series of visualizations (Figures 3–8) explores univariate exposure–response relationships, quantifies interactions among exposures, and characterizes overall and quantile-specific effects of individual chemicals. These analyses, adjusted for key demographic, anthropometric, and lifestyle covariates, enable a comprehensive evaluation of both individual and joint effects of chemical exposures on reproductive hormone regulation.

For estradiol, BKMR analyses revealed a predominantly flat univariate exposure–response landscape with a small number of chemicals showing notable individual signals, and a monotonically increasing overall mixture effect (Figure 3). In the univariate exposure–response functions (Figure 3A), nearly all chemicals showed flat associations near zero when other exposures were held at their median. The only exception with any notable pattern was o-xylene, which showed a dramatic spike at very low exposure concentrations but with extremely wide credible intervals, indicating substantial uncertainty; all other chemicals, including PFASs, metals, and phthalate metabolites, displayed negligible univariate associations with estradiol. Single-variable summary results (Figure 3B) revealed a different picture when comparing effects across the interquartile range: lead showed the strongest and most clearly negative association (estimate approximately −80, credible interval clearly excluding zero), indicating that higher lead exposure relative to the mixture background is associated with lower estradiol. PFOS also showed a negative estimate, though the credible interval approached zero. By contrast, o-xylene and benzene showed positive single-variable associations. Most other chemicals clustered tightly near zero with narrow credible intervals. Single-exposure-attributable effects (Figure 3C) confirmed that lead exerted the dominant individual contribution, with increasing lead from the 25th to 75th percentile associated with a large negative change in estradiol that was consistent across all three background mixture levels, indicating a genuine independent suppressive effect. PFOS showed a modest positive attributable effect. All other chemicals produced effects indistinguishable from zero. The overall mixture effect (Figure 3D) showed a monotonically increasing dose–response pattern, with combined exposures at the 25th percentile associated with estradiol suppression of approximately −10, transitioning through zero near the 55th–60th percentile, and reaching approximately +20 at the 75th percentile. Although 95% credible intervals were wide and encompassed zero throughout much of the exposure range, the consistent directionality of the pattern suggests that low combined exposures are associated with estradiol suppression while higher combined exposures are associated with estradiol elevation.

For LH, BKMR analyses revealed a small number of chemicals with notable individual patterns and a monotonically decreasing overall mixture effect (Figure 4). Univariate exposure–response functions (Figure 4A) showed flat curves for most exposures when other chemicals were held at their median. Lead was the primary exception, exhibiting a non-monotonic pattern that rose and then declined as concentration increased, accompanied by credible bands indicating moderate uncertainty. PFOA showed a declining trend at low exposure concentrations. MBzP showed a volatile, noisy positive pattern at very low concentrations with wide credible intervals. o-Xylene showed an initial sharp spike at low exposure then flattened. All remaining phthalate metabolites and VOCs showed negligible univariate associations.

Single-variable summary results (Figure 4B) showed that MBzP had the strongest positive single-variable association with LH (estimate approximately +0.6, credible interval clearly excluding zero), indicating that higher MBzP relative to the mixture background is associated with higher LH. Lead also showed a positive estimate with a wide credible interval. Mercury showed a slight negative estimate. All remaining chemicals clustered tightly near zero with narrow credible intervals, indicating negligible mixture–context-dependent effects on LH for the vast majority of exposures.

Single-exposure-attributable effects (Figure 4C) revealed that MBzP was the primary individual exposure of concern, with increasing MBzP from the 25th to 75th percentile associated with a consistent negative change in LH of approximately −3 to −4 units across all three background mixture levels. The consistency of this negative attributable effect across the 25th, 50th, and 75th percentile backgrounds indicates a genuine independent suppressive contribution. This attributable suppression of LH by MBzP contrasts with its positive single-variable association in Figure 4B, suggesting that MBzP’s observed positive association in that panel reflects the context of elevated co-exposures rather than a direct independent effect. Lead, PFOS, and PFOA showed very small positive attributable effects near zero with overlapping credible intervals across background levels. All other chemicals produced effects indistinguishable from zero.

The overall mixture effect (Figure 4D) showed a monotonically decreasing dose–response pattern, with combined exposures at the 25th percentile associated with a modest LH elevation of approximately +1.2, declining progressively through zero near the 50th percentile, and reaching approximately −1.1 at the 75th percentile. The credible intervals were relatively narrow throughout, indicating reasonable certainty in this monotonically declining pattern. This consistent dose–response suggests that increasing cumulative chemical exposures are associated with progressive LH suppression, with lower combined exposures associated with modest stimulation and higher combined exposures associated with clear suppression of luteinizing hormone.

For SHBG, BKMR analyses revealed heterogeneous associations between environmental chemical exposures and SHBG, with notable individual contributors identified in each chemical class (Figure 5). Univariate exposure–response functions (Figure 5A) showed that most phthalate metabolites and metals exhibited relatively flat response curves, indicating negligible univariate associations across the exposure distribution when other chemicals were held at their median values. MEHP showed a visually pronounced positive pattern at very high exposure levels, but this was accompanied by wide credible bands and is not supported by the formal PIP analysis (condPIP = 0.0 for SHBG). PFOA, benzene, o-xylene, and mp-xylene showed slight negative trends, though effect magnitudes remained modest throughout.

Single-variable summary results (Figure 5B) showed that benzene had the strongest positive single-variable association with SHBG (estimate approximately +0.3, credible interval clearly positive), indicating that benzene’s effect on SHBG is amplified within the mixture context. MEHP also showed a positive estimate with a wide credible interval. Cadmium showed a clearly negative single-variable association, indicating its effect on SHBG is suppressive within the mixture context. All other chemicals clustered near zero with narrow credible intervals.

Single-exposure-attributable effects (Figure 5C) confirmed that MEHP had the dominant independent contribution to SHBG regulation. Increasing MEHP from the 25th to 75th percentile was associated with a large positive change in SHBG of approximately +5 to +6, consistent across all three background mixture quantile levels, confirming a genuine independent effect rather than a mixture-driven artifact. PFOA showed a modest positive attributable effect. Cadmium showed a negative attributable effect. All remaining chemicals produced effects near zero with overlapping credible intervals across background quantile levels.

The overall mixture effect (Figure 5D) showed a monotonically increasing dose–response pattern, with combined exposures at the 25th percentile associated with SHBG suppression of approximately −1.8, rising progressively through zero near the 50th percentile, and reaching approximately +3.2 at the 75th percentile. The credible intervals were moderately wide but consistently captured the zero line only in the lower-to-mid exposure range, with the upper quantiles showing estimates clearly above zero. This monotonic increase suggests that the cumulative effect of the chemical mixture shifts SHBG from suppression to elevation with increasing overall exposure, consistent with the large positive attributable effect of MEHP identified in Figure 5C.

For FSH, BKMR analyses revealed predominantly null univariate associations between individual chemical exposures and FSH, with select chemicals demonstrating notable nonlinear patterns, and a pronounced biphasic overall mixture effect (Figure 6). Univariate exposure–response functions (Figure 6A) showed flat associations for most chemicals across the observed exposure distribution. PFOA was the most distinctive exception, exhibiting a non-monotonic pattern with FSH elevation at very low exposure levels followed by progressive suppression at higher concentrations, suggestive of a hormetic-type response. MCOP showed a modest positive association at lower exposures that plateaued at higher concentrations. Volatile organic compounds, including dichlorobenzene, showed an upward trend, while o-xylene and mp-xylene exhibited slight U-shaped or otherwise nonlinear patterns, though effect magnitudes remained small throughout. Most remaining Phthalate metabolites and metals displayed negligible univariate associations with FSH.

Single-variable summary results (Figure 6B) identified two chemicals with notable single-variable associations for FSH. PFOA demonstrated a positive single-variable association (estimate approximately +0.3, though credible interval clearly including zero), indicating that PFOA’s effect on FSH is amplified within the mixture context. MCOP also showed a positive estimate with a wide credible interval, suggesting modest mixture–context-dependent positive effects. Mercury showed the strongest negative single-variable association (estimate approximately −0.5), indicating that mercury’s effect on FSH within the mixture context is suppressive. Cadmium also showed a slight negative estimate. All remaining chemicals clustered tightly near zero with narrow credible intervals.

Single-exposure-attributable effects (Figure 6C) confirmed that PFOA and MCOP had the dominant positive independent contributions to FSH regulation. Increasing PFOA from the 25th to 75th percentile was associated with a large positive change in FSH of approximately +5 to +7, consistent across all three background mixture quantile levels. MCOP also showed a positive, meaningful attributable effect. Mercury showed a clearly negative attributable effect, confirming its independent suppressive contribution to FSH independently of co-exposure levels. All remaining chemicals produced effects near zero.

The overall mixture effect (Figure 6D) showed the most pronounced and consistently monotonically increasing dose–response pattern observed across all hormones examined. At lower mixture concentrations (25th–40th percentiles), combined exposures were associated with substantial FSH suppression, with estimates ranging from approximately −4.5 at the 25th percentile to −2.5 at the 40th percentile. The effect transitioned through zero near the 45th–50th percentile, followed by progressive and substantial FSH elevation at higher mixture levels, with estimates increasing to approximately +6.5 at the 75th percentile. The credible intervals were relatively narrow throughout, particularly around the transition zone, indicating greater statistical confidence in the direction and magnitude of this effect compared to other hormones. The smooth monotonic progression from suppression to elevation across the full mixture quantile range indicates a strong and consistent dose-dependent relationship between cumulative chemical exposure and FSH.

For AMH, BKMR analyses revealed predominantly null univariate associations but notable individual chemical signals in the single-variable and attributable effect panels, and a monotonically increasing overall mixture effect (Figure 7). Univariate exposure–response functions (Figure 7A) demonstrated near-zero associations for most chemicals. MBzP showed a volatile, noisy positive pattern at very low concentrations with very wide credible bands, indicating high uncertainty in its univariate relationship. Mercury showed a clear non-monotonic (inverted-U) curve, peaking at intermediate concentrations and declining thereafter. PFOA showed a slight dip at low concentrations before returning to zero. Benzene showed a very slight non-monotonic pattern at very low concentrations. All remaining chemicals displayed flat response curves near zero. Single-variable summary results (Figure 7B) revealed that mercury had the strongest and most clearly positive single-variable association with AMH (estimate approximately +0.2, credible interval clearly excluding zero), indicating that mercury’s effect on AMH is consistently positive within the mixture context. MBzP showed a wide credible interval crossing zero. Benzene showed a slightly negative estimate. PFOA showed a negative estimate. All other chemicals clustered tightly near zero. Single-exposure-attributable effects (Figure 7C) confirmed that MBzP had the dominant positive independent contribution to AMH, with increasing MBzP from the 25th to 75th percentile associated with a positive change of approximately +0.8 to +1.0, consistent across all background mixture levels. PFOA showed a clearly negative attributable effect (approximately −0.5), indicating independent suppression of AMH. Mercury showed a modest positive attributable effect. Benzene showed a modest negative attributable effect. All other chemicals produced effects near zero.

The overall mixture effect (Figure 7D) showed a monotonically increasing pattern, with combined exposures at the 25th percentile associated with modest AMH suppression of approximately −0.10, rising progressively through zero near the 50th percentile, and reaching approximately +0.30 at the 75th percentile. The credible intervals although wide, did not overlap zero throughout much of the lower exposure range, indicating that the suppressive effects at low mixture concentrations are uncertain. However, the consistent directional increase across the full quantile range is notable. Effect magnitudes were substantially smaller than those observed for FSH, progesterone, and estradiol, reflecting AMH’s distinct biology as a marker of ovarian reserve that integrates hormonal status over a longer time horizon than the other reproductive hormones examined.

For progesterone, BKMR analyses showed a stark contrast between the richness of individual chemical associations in linear regression (Section 3.2.6) and the near-complete flatness of BKMR univariate curves, with the overall mixture effect showing a large but highly uncertain monotonically increasing pattern (Figure 8). Univariate exposure–response functions (Figure 8A) showed that virtually all chemicals had completely flat associations near zero when other exposures were held at their median. The sole exception was MHIBP, which exhibited a sharp positive spike at very high exposure concentrations, but this was accompanied by very wide credible bands reflecting extreme uncertainty at those concentrations. This near-universal flatness across all chemical classes indicates that individual chemical effects on progesterone are almost entirely dependent on co-exposure context when assessed through the BKMR kernel function.

Single-variable summary results (Figure 8B) showed that the vast majority of chemicals had point estimates tightly clustered at zero with very narrow credible intervals, confirming negligible mixture–context-dependent effects for most exposures. Three chemicals stood out by having the widest credible intervals, though all crossed zero: MHIBP (point estimate approximately −55, very wide CI), PFOA (point estimate approximately −100, very wide CI), and cadmium (point estimate near zero, wide CI). The extreme scale of the credible intervals for MHIBP and PFOA in this panel reflects high uncertainty rather than confirmed direction of effect, and these estimates should be interpreted with caution.

Single-exposure-attributable effects (Figure 8C) showed that PFOA had the most notable attributable effect on progesterone, with a negative point estimate of approximately −100 and wide credible intervals, though the CIs crossed zero indicating substantial uncertainty. MHIBP showed a positive attributable point estimate but with equally wide credible intervals crossing zero (except the 75th percentile). Cadmium showed a modest positive attributable effect. All remaining chemicals produced effects near zero with overlapping colored points across the three background quantile levels, indicating an absence of meaningful independent individual effects on progesterone for the vast majority of chemicals.

Despite the flat individual chemical curves in panels A through C, the overall mixture effect (Figure 8D) revealed the largest magnitude effect observed across all hormones, though also the greatest uncertainty. The overall mixture effect was monotonically increasing, with combined exposures at the 25th percentile associated with progesterone suppression of approximately −35, rising through zero near the 50th percentile, and reaching approximately +10 at the 75th percentile. However, the credible intervals were extremely wide, particularly at the extremes: the lower credible interval at the 25th percentile extends to approximately −90, while the upper credible interval at the 75th percentile extends to approximately +50. This extreme width indicates that while the direction of the overall mixture effect is consistent, the absolute magnitude is highly uncertain. The contrast between the flat individual chemical curves and the large overall mixture effect strongly suggests that progesterone sensitivity to the chemical mixture is almost entirely an emergent property of the joint exposure distribution, rather than being driven by any single chemical’s independent contribution.

## 4. Discussion

### 4.1. Overview and Key Findings

This study provides a comprehensive, mixture-based assessment of the associations between four major classes of environmental endocrine-disrupting chemicals (EDCs), per- and polyfluoroalkyl substances (PFASs), toxic metals, phthalates, and volatile organic compounds (VOCs) and six key reproductive hormones: estradiol, follicle-stimulating hormone (FSH), luteinizing hormone (LH), progesterone, sex-hormone-binding globulin (SHBG), and anti-Müllerian hormone (AMH). Leveraging the nationally representative NHANES 2017–2018 data and employing both linear regression and the advanced Bayesian Kernel Machine Regression (BKMR) methodology, our analysis revealed complex, nonlinear, and interactive alterations in reproductive endocrine function. The central finding is the clear demonstration of significant mixture effects, which underscores the inadequacy of traditional single-pollutant models in capturing real-world human exposure and risk [51]. The BKMR approach was instrumental in providing a nuanced understanding of joint toxicity, non-monotonic dose–response curves, and the relative importance of individual mixture components via Posterior Inclusion Probabilities (PIPs) [52]. The minimal cross-class correlations (r < 0.2) suggest that the four chemical classes represent largely independent exposure domains, reinforcing the need for a mixture-based approach to capture their combined biological impact. All findings reported in this study are statistical associations; the cross-sectional design precludes causal inference and cannot establish temporal precedence. Reverse causation, whereby altered endogenous hormone concentrations influence the metabolism and measured biomarker levels of environmental chemicals, cannot be excluded and should be considered when interpreting results.

### 4.2. Linear Regression Findings

Linear regression analyses revealed extensive heterogeneous associations between the environmental chemicals examined and reproductive hormones, with statistically significant findings across all four chemical classes for most hormone endpoints. For estradiol, the positive association with lead and inverse association with mercury are consistent with previous studies linking heavy metal exposure to altered steroidogenesis and HPG axis disruption [17,48]. The heterogeneous associations observed for phthalate metabolites with estradiol (positive for MCNP, MBP, MCOCH, MEHHP, and MHHT; inverse for MCPP, MHNCH, MEHP, and MHBP, with MONP showing a nominally significant inverse association that did not survive FDR correction) and for VOCs (positive for dichlorobenzene; benzene and mp-xylene showed nominally significant associations that did not survive FDR correction) reflect the diverse mechanisms of action within and across these classes. This finding is particularly noteworthy as it challenges the assumption of a uniform effect for all phthalates, suggesting that specific metabolites may differentially interfere with estrogen synthesis or metabolism, a complexity noted in toxicological reviews [53].

The strong and extensive positive associations between numerous chemical exposures and SHBG—spanning both PFASs (PFOA and PFOS), multiple phthalate metabolites (MCNP, MECP, MHHT, MHNCH, MEHP, MEOHP, MONP, and MBzP), and VOCs (mpxylene and dichlorobenzene)—are consistent with prior epidemiological findings [54,55]. This pattern suggests that many EDCs may induce a compensatory upregulation of SHBG, potentially reflecting a hepatic response to endocrine disruption or an alteration in hormone transport protein synthesis [56]. However, this finding contrasts with some literature that reports inverse associations between high levels of certain phthalate metabolites and SHBG [57]. This discrepancy highlights the importance of exposure level and chemical-specific effects, suggesting that while some EDCs may directly suppress SHBG synthesis, others may trigger a systemic compensatory mechanism. Contrasting patterns for progesterone were found across all four chemical classes, with multiple inverse associations (PFOA, cadmium, lead, MHIBP, MEHHP, MEHP, MCOP, ECPT, and MCOCH, the last of which was nominally significant only) and positive associations (PFOS, mercury, MCNP, MECP, MHHT, MEOHP, MONP, MBzP, and benzene). This underscores the complexity of EDC mechanisms affecting reproductive steroid synthesis. Similarly, AMH demonstrated extensive heterogeneous associations spanning all chemical classes, with inverse associations for PFOA, cadmium, and lead, and a nominally significant inverse association for MHIBP that did not survive FDR correction, and positive associations for PFOS, mercury, MCNP, MCPP, MEHHP, MHHT, MHBP, MiBP, and benzene, providing substantial evidence of EDC impact on ovarian reserve markers warranting further investigation.

### 4.3. Posterior Inclusion Probabilities (PIPs): Identifying Key Drivers

Posterior inclusion probabilities provide critical insights into which environmental chemicals are most relevant for predicting each reproductive hormone outcome. For estradiol, the conditional PIPs pinpointed cadmium (1.0) and the phthalate metabolite MBzP (1.0) as the dominant metal and phthalate predictors, respectively, with PFOS also showing a conditional PIP of 1.0 for estradiol. This suggests that while the overall mixture is important, a small number of highly potent EDCs may drive the majority of the effect on estradiol. The selective patterns for FSH and LH further emphasize this point. PFOA was the dominant PFAS predictor for FSH (conditional PIP = 1.0), with lead also showing near-maximal conditional importance (PIP = 0.9956), while cadmium (0.9772) and MBzP (1.0) maintained high conditional importance for LH, along with mp-xylene as the dominant VOC predictor (conditional PIP = 1.0). The high conditional importance of MHIBP for progesterone (1.0) and MEHP for SHBG (1.0) aligns with the known anti-androgenic and metabolic disruptive properties of these specific phthalate metabolites [53]. For AMH, PFOA, lead, and MBzP each achieved conditional PIPs of 1.0, making them the top individual predictors despite the high uncertainty in overall AMH mixture effects. For SHBG, benzene emerged as a notable VOC predictor with a conditional PIP of 0.9333, alongside PFOA (1.0) and MEHP (1.0). These PIP results provided a quantitative basis for identifying the most critical mixture components for regulatory and mechanistic investigation, allowing for targeted risk assessment.

### 4.4. BKMR Results and Exposure–Response Relationship

The BKMR analysis provided a detailed characterization of the exposure–response functions, revealing non-monotonicity and chemical-chemical interactions that are critical for understanding real-world risk.

#### 4.4.1. Estradiol

BKMR analyses for estradiol identified lead as the dominant individual contributor, with a strongly negative single-variable association and a large negative attributable effect (approximately −40 to −80 units). PFOS also showed a negative single-variable association. By contrast, o-xylene and benzene showed positive single-variable associations. Contrary to previously stated descriptions, DEHP metabolites (MEHP, MEOHP, MEHHP) showed essentially flat univariate curves near zero when other exposures were held at their median. The overall mixture effect was monotonically increasing across the quantile range, from approximately −10 at the 25th percentile to approximately +20 at the 75th percentile, crossing zero near the 55th–60th percentile. Although credible intervals were wide throughout, the consistent direction from suppression to elevation across the full mixture distribution is consistent with an exposure-dependent shift in estrogenic balance [58].

#### 4.4.2. Luteinizing Hormone (LH)

Univariate exposure–response functions for LH were generally flat when other exposures were held at their median, with lead showing a non-monotonic curve and MBzP showing a volatile pattern at low concentrations. MBzP was the dominant individual contributor across panels, with the strongest positive single-variable association and the largest negative attributable effect (−3 to −4 units), indicating that MBzP’s effect on LH is strongly dependent on co-exposure context. The overall mixture effect showed a monotonically decreasing pattern: LH was elevated at the 25th percentile (approximately +1.2), declining progressively through zero near the 50th percentile, and reaching approximately −1.1 at the 75th percentile, with relatively narrow credible intervals throughout. This consistent monotonic decline indicates that increasing cumulative chemical exposure is associated with progressive LH suppression.

#### 4.4.3. Sex-Hormone-Binding Globulin (SHBG)

SHBG BKMR analyses identified MEHP as the dominant phthalate contributor (large positive attributable effect ~+5 to +6), benzene as the strongest positive single-variable contributor, and cadmium as having a negative single-variable and attributable effect. The univariate curve for MEHP clearly rises at higher concentrations; MHIBP showed a visually pronounced univariate pattern but its conditional PIP for SHBG is 0.0, indicating it is not a confirmed independent driver. The overall mixture effect showed a monotonically increasing dose–response pattern, transitioning from SHBG suppression at lower mixture levels (approximately −1.8 at the 25th percentile) through zero near the 50th percentile and rising to approximately +3.2 at the 75th percentile, consistent with the dominant positive contribution of MEHP. This pattern may reflect progressive induction of hepatic SHBG synthesis at higher combined exposures [48,56].

#### 4.4.4. Follicle-Stimulating Hormone (FSH)

FSH BKMR analyses identified PFOA and MCOP as the dominant positive individual contributors, and mercury as the dominant negative contributor, based on the attributable effects analysis. PFOA showed a hormetic-type inverted-U univariate pattern and the largest positive attributable effect (approximately +5 to +7 units). MCOP showed a similar inverted-U univariate pattern and a positive attributable effect. Mercury, by contrast, showed a consistently negative single-variable association and a clearly negative attributable effect, indicating independent suppression of FSH. The overall mixture effect revealed the most pronounced and monotonically increasing dose–response pattern observed across all hormones examined, transitioning from substantial suppression (approximately −4.5 at the 25th percentile) through zero near the 45th–50th percentile, and rising to approximately +6.5 at the 75th percentile, with relatively narrow credible intervals throughout. This striking response for a pituitary gonadotropin challenges the assumption of linearity in traditional toxicological models [59].

#### 4.4.5. Anti-Müllerian Hormone (AMH)

AMH BKMR analyses identified a distinct pattern of individual contributors. Mercury had the strongest positive single-variable association (Figure 7B, estimate clearly positive). MBzP had the dominant positive attributable effect (+0.8 to +1.0, Figure 7C). PFOA had a clearly negative attributable effect (−0.5, Figure 7C), indicating independent suppression. Benzene showed a modest negative attributable effect. The overall mixture effect was monotonically increasing, with suppression at lower mixture concentrations (approximately −0.10 at the 25th percentile) transitioning through zero near the 50th percentile and rising to approximately +0.30 at the 75th percentile, though credible intervals were wide throughout. Effect magnitudes were substantially smaller than those observed for FSH, progesterone, and estradiol. Interpretation of these AMH findings requires important contextual caveats. AMH reference ranges differ substantially by sex and age: in females, AMH peaks in the mid-20 s and declines progressively with age, reaching undetectable levels post-menopause, while in males, AMH is high in childhood and declines following puberty. Analyzing AMH across the full NHANES age spectrum without stratification therefore introduces substantial physiological heterogeneity that likely attenuates observed associations and limits interpretability. These age- and sex-driven differences are partially accounted for through covariate adjustment, but residual confounding remains a meaningful limitation. Additionally, the cross-sectional NHANES design may be insufficient to capture the longer-term ovarian reserve dynamics that AMH reflects, suggesting that prospective studies with age- and sex-stratified AMH analyses are needed.

#### 4.4.6. Progesterone

BKMR univariate exposure–response curves for progesterone were almost entirely flat, with MHIBP being the sole exception showing a dramatic spike at very high concentrations with wide credible bands. PFOA showed the most notable attributable effect among individual chemicals (negative, approximately −100 units, but with very wide credible intervals crossing zero). This near-universal individual flatness contrasts with the numerous significant associations identified in multivariable linear regression (Section 3.2.6), indicating that progesterone’s individual chemical responses are almost entirely dependent on co-exposure context. The overall mixture effect was monotonically increasing, with estimates ranging from approximately −35 at the 25th percentile to approximately +10 at the 75th percentile, but with extremely wide credible intervals throughout. These values represent model-estimated differences in progesterone concentrations in ng/dL. The magnitude of this overall mixture effect far exceeds any individual chemical estimate, confirming that progesterone’s response to this chemical mixture is an emergent joint property, not predictable from single-chemical analyses.

### 4.5. Strengths and Limitations

#### 4.5.1. Strengths

The primary strength of this study is the use of the BKMR methodology, which is a robust statistical tool for assessing the health effects of complex environmental mixtures, allowing for the simultaneous evaluation of nonlinear exposure–response functions and exposure-exposure interactions. Furthermore, the use of the nationally representative NHANES data provides high external validity, ensuring that the findings are generalizable to the noninstitutionalized U.S. population. The comprehensive panel of 28 EDCs across four major classes and six reproductive hormones provides an unparalleled view of endocrine disruption in a real-world exposure scenario.

#### 4.5.2. Limitations

This study is subject to the following limitations. First, the cross-sectional design of the NHANES data precludes establishing causal relationships, limiting our findings to associations. Second, the use of a single spot urine or blood sample for chemical measurements may not accurately reflect long-term or critical-window exposures, particularly for chemicals with short biological half-lives, such as phthalates and VOC metabolites. Third, AMH results should be interpreted with particular caution given the substantial age- and sex-related variation in this biomarker across the full NHANES age spectrum; future studies should analyze AMH in age- and sex-stratified subgroups. Fourth, the study focused exclusively on measured EDCs; unmeasured co-exposures and other lifestyle factors may contribute to residual variance in hormone levels. Future prospective studies are warranted to confirm the temporal relationships observed here. Fifth, exposure variables were derived from different biological matrices (serum, whole blood, urine) with differing pharmacokinetic half-lives, limiting direct comparability and temporal alignment across chemical classes; phthalate and VOC measurements reflect short-term exposure windows. Sixth, sex-stratified BKMR analyses were not conducted due to limited statistical power; future studies should implement sex-specific mixture analyses. Seventh, BKMR does not natively support complex survey weights, so BKMR estimates may not be fully representative of the U.S. population in the same manner as survey-weighted linear regression estimates. Eighth, all findings are associations only; the cross-sectional design precludes assessment of temporality, and reverse causation cannot be excluded. Ninth, while the MAR assumption underlying the MICE procedure is supported by the design-driven nature of the dominant source of missingness, item nonresponse in NHANES may in some instances be missing not at random (MNAR). Individuals with higher environmental exposure burdens or poorer health status may be less likely to provide biological specimens, potentially introducing systematic bias that cannot be detected or corrected under the MAR framework. Formal MNAR sensitivity analyses were not feasible given the complexity of the 28-variable exposure dataset; however, complete-case analyses yielded directionally consistent results, reassuring that MNAR bias, if present, is unlikely to substantially alter the study’s conclusions.

## 5. Conclusions

Our study showed that exposure to real-world mixtures of PFASs, toxic metals, phthalates, and VOCs are associated with measurable alterations in reproductive hormone levels. Using both linear regression and BKMR, we identified pronounced nonlinear, non-monotonic dose–response relationships across all six hormones, with MEHP, cadmium, PFOA, and MBzP emerging as key individual contributors identified by the BKMR posterior inclusion probabilities. Estradiol, FSH, LH, and progesterone demonstrated the most robust non-monotonic mixture-level dose–response patterns; SHBG showed a directionally consistent biphasic pattern with substantial uncertainty, while AMH associations were largely uncertain given wide credible intervals and the inherent age- and sex-related heterogeneity of this biomarker across the full NHANES age spectrum. Together, these findings indicate that risk does not increase in a simple linear fashion with exposure. These findings reinforce the need for mixture-based approaches in environmental health research, as single-chemical models may underestimate or mischaracterize reproductive risks. Future longitudinal studies are needed to clarify temporality, identify critical windows of susceptibility, and improve the mechanistic understanding of mixture-driven endocrine disruption.

## Figures and Tables

**Figure 1.**
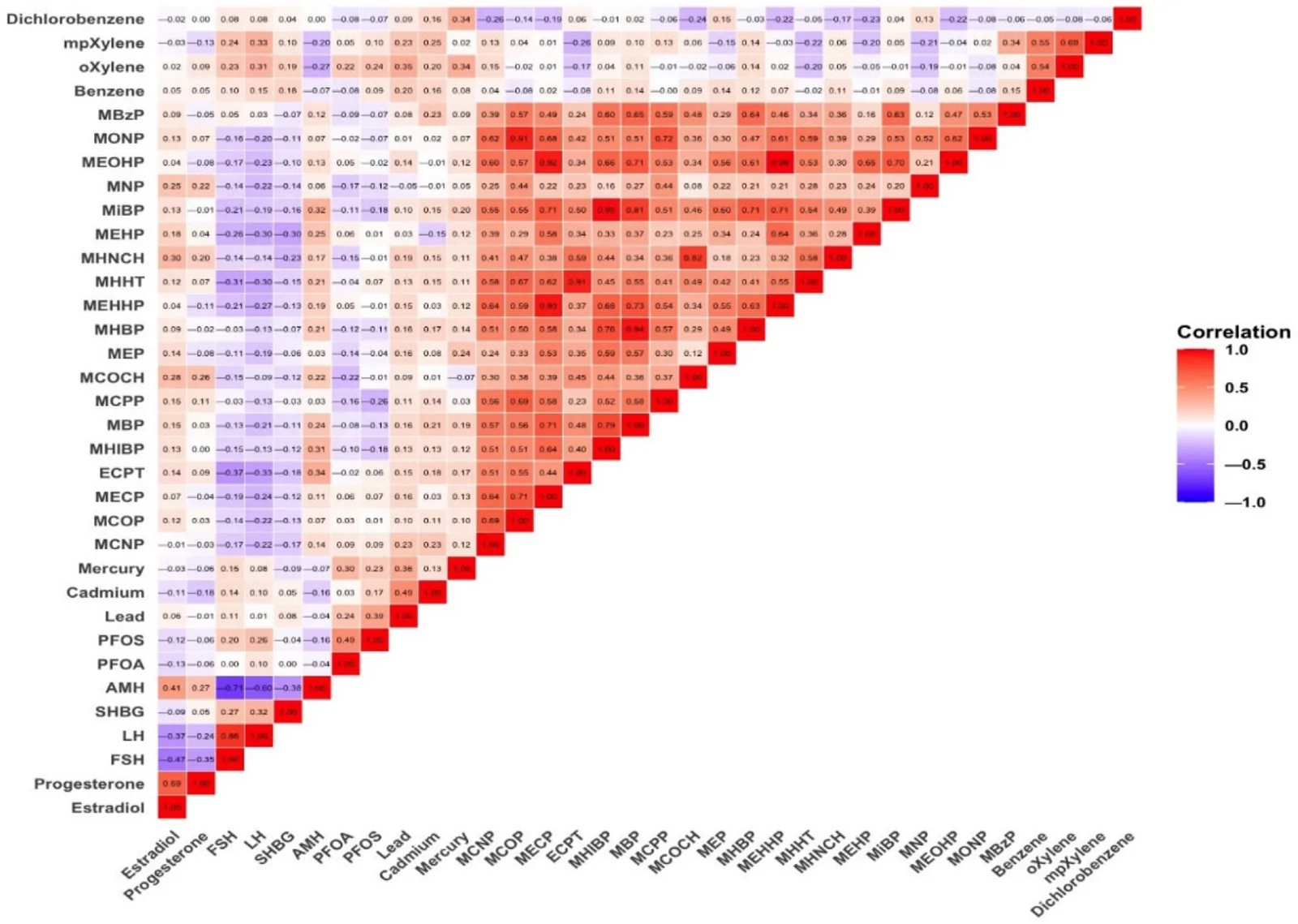
Spearman correlation matrix of reproductive hormones and environmental exposures.

**Figure 2.**
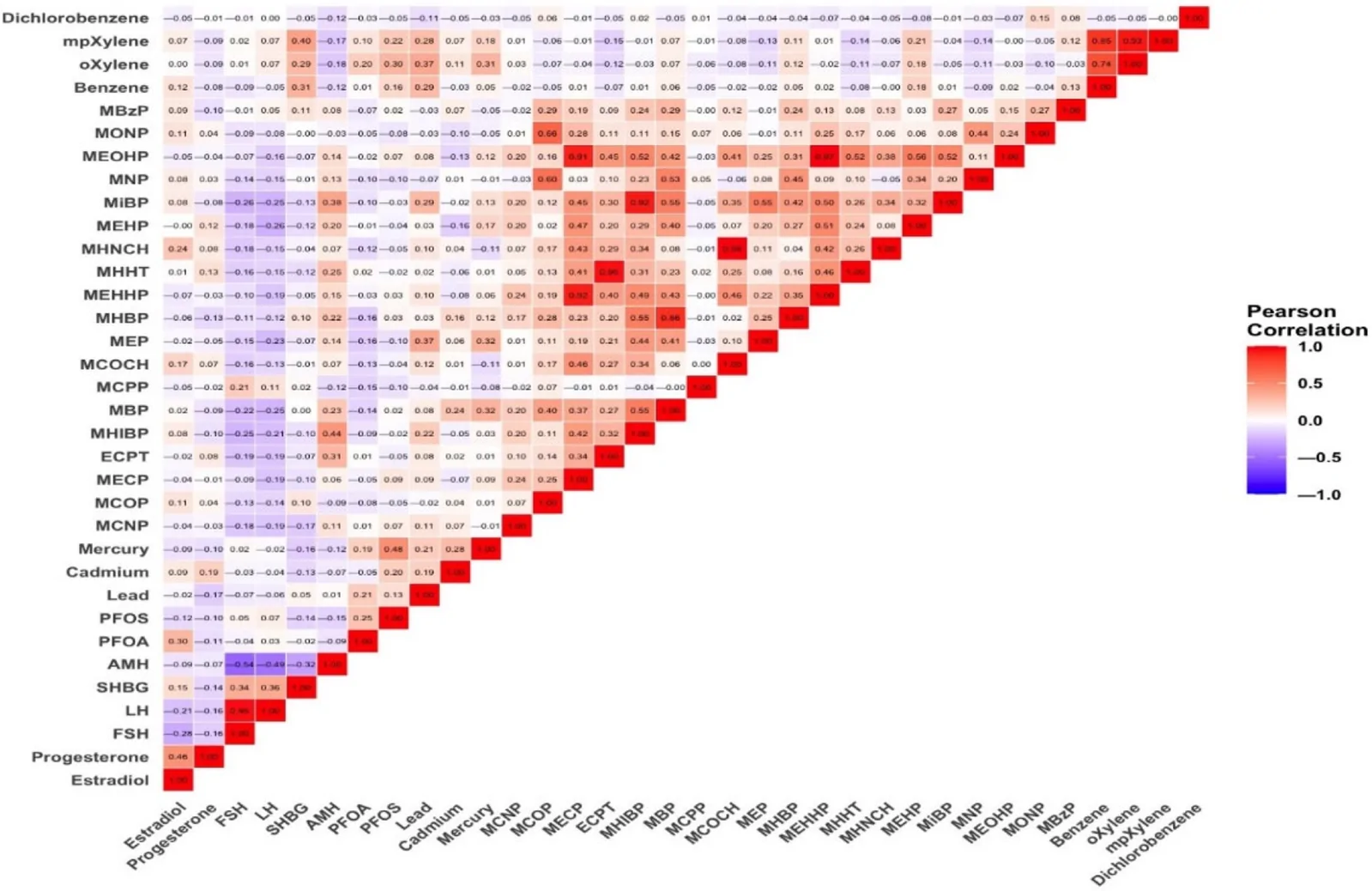
Pearson correlation matrix of reproductive hormones and environmental exposures.

**Figure 3.**
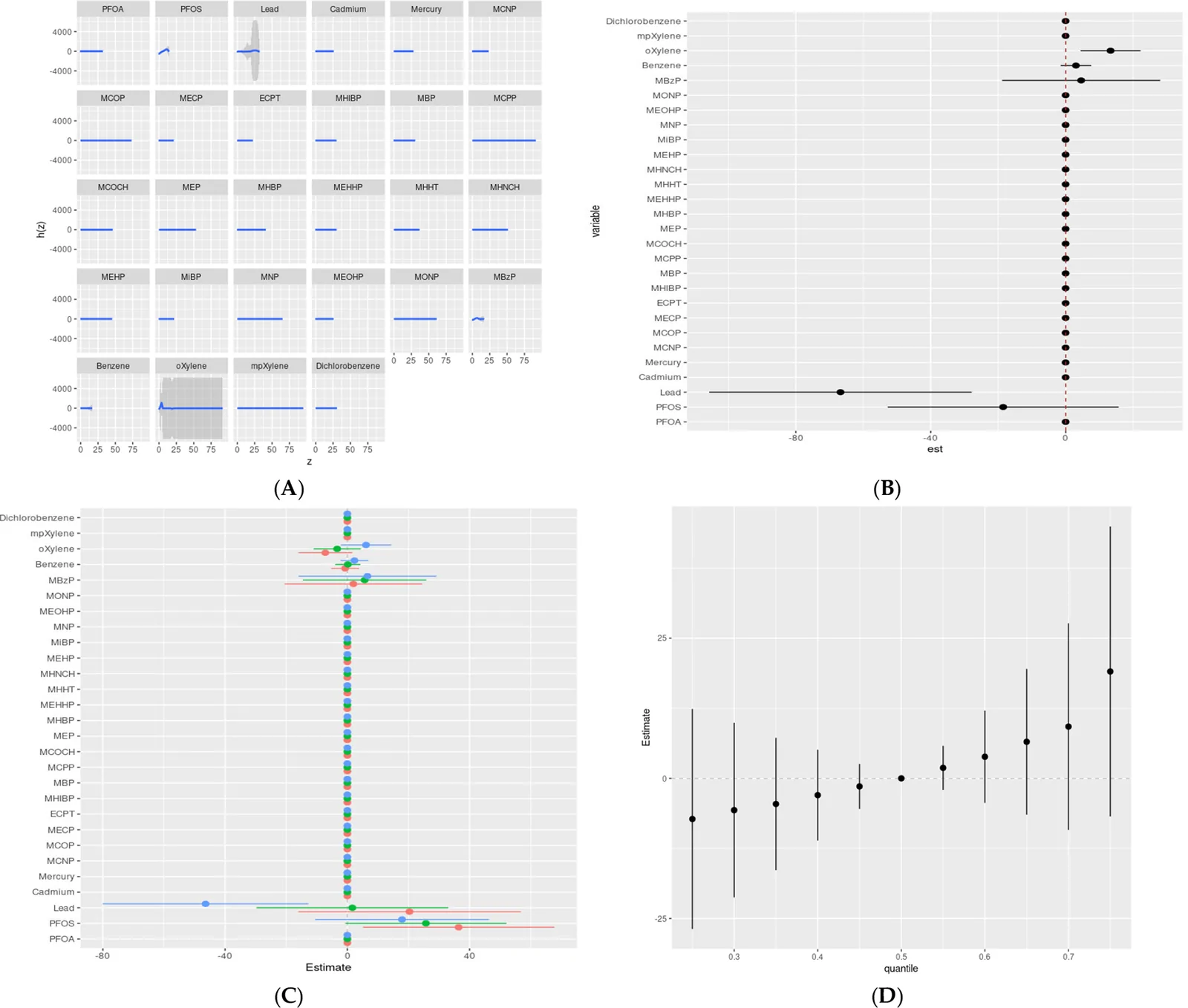
BKMR results for estradiol. (**A**) Univariate exposure–response functions with 95% credible intervals illustrating associations between individual chemical exposures and estradiol concentrations, with other exposures fixed at median values. (**B**) Single-variable summary of the effect of each exposure on estradiol, comparing the 75th to the 25th percentile while all other exposures are fixed at their median. (**C**) Expected change in estradiol associated with increasing each exposure from the 25th to 75th percentile, evaluated when all other exposures are fixed at the 25th (red), 50th (green), or 75th (blue) percentile of the mixture. (**D**) Overall mixture effects and 95% credible intervals for combined chemical exposures on estradiol across mixture quantiles from the 25th to 75th percentiles. Analyses are adjusted for age, sex, race/ethnicity, BMI, smoking, alcohol consumption, diabetes, annual income, hypertension, and steroid use.

**Figure 4.**
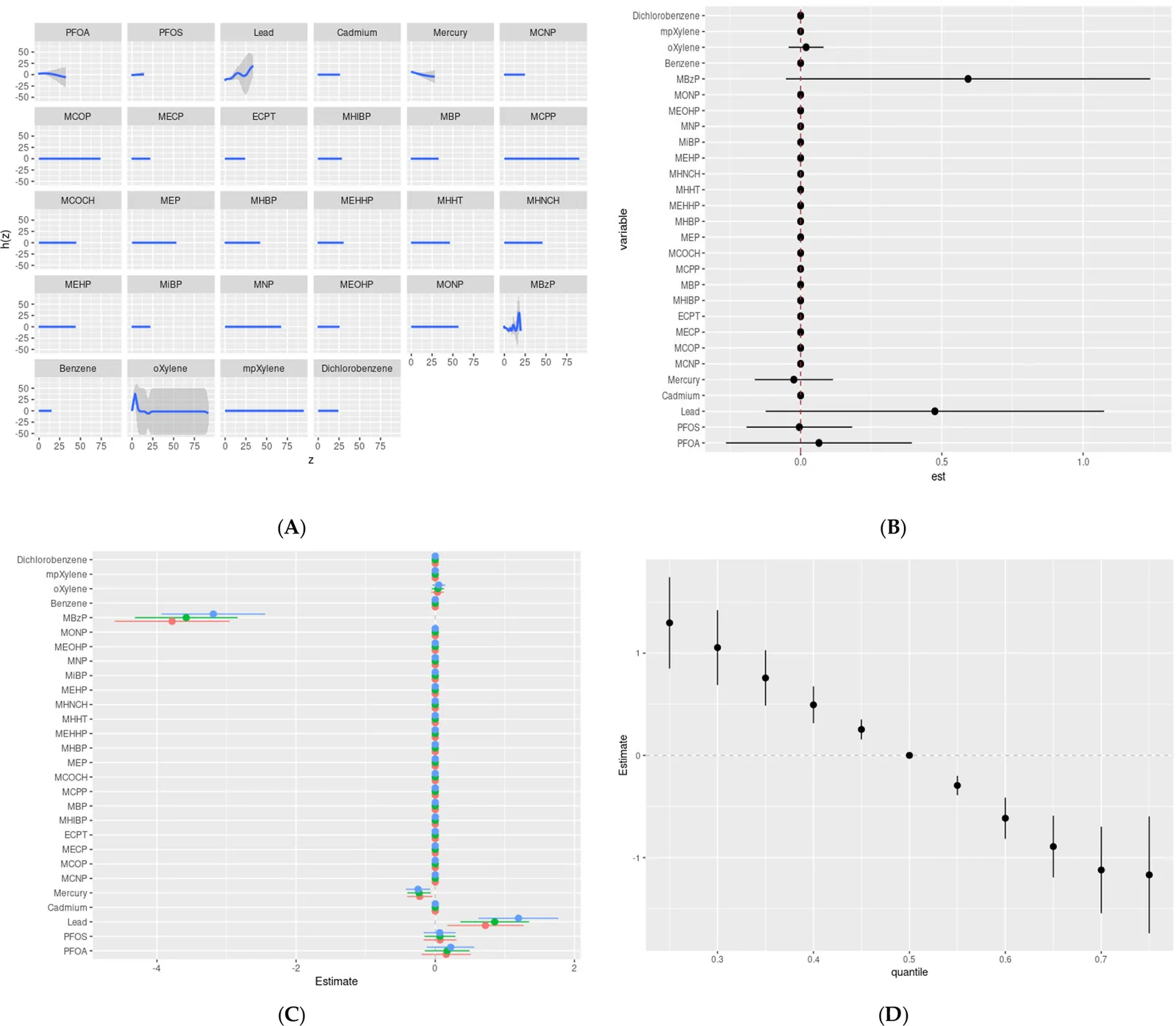
BKMR results for luteinizing hormone. (**A**) Univariate exposure–response functions with 95% credible intervals illustrating associations between individual chemical exposures and LH concentrations, with other exposures fixed at median values. (**B**) Single-variable summary of the effect of each exposure on LH, comparing the 75th to the 25th percentile while all other exposures are fixed at their median. (**C**) Expected change in LH associated with increasing each exposure from the 25th to 75th percentile, evaluated when all other exposures are fixed at the 25th (red), 50th (green), or 75th (blue) percentile of the mixture. (**D**) Overall mixture effects and 95% credible intervals for combined chemical exposures on LH across mixture quantiles from the 25th to 75th percentiles. Analyses are adjusted for age, sex, race/ethnicity, BMI, smoking, alcohol consumption, diabetes, annual income, hypertension, and steroid use.

**Figure 5.**
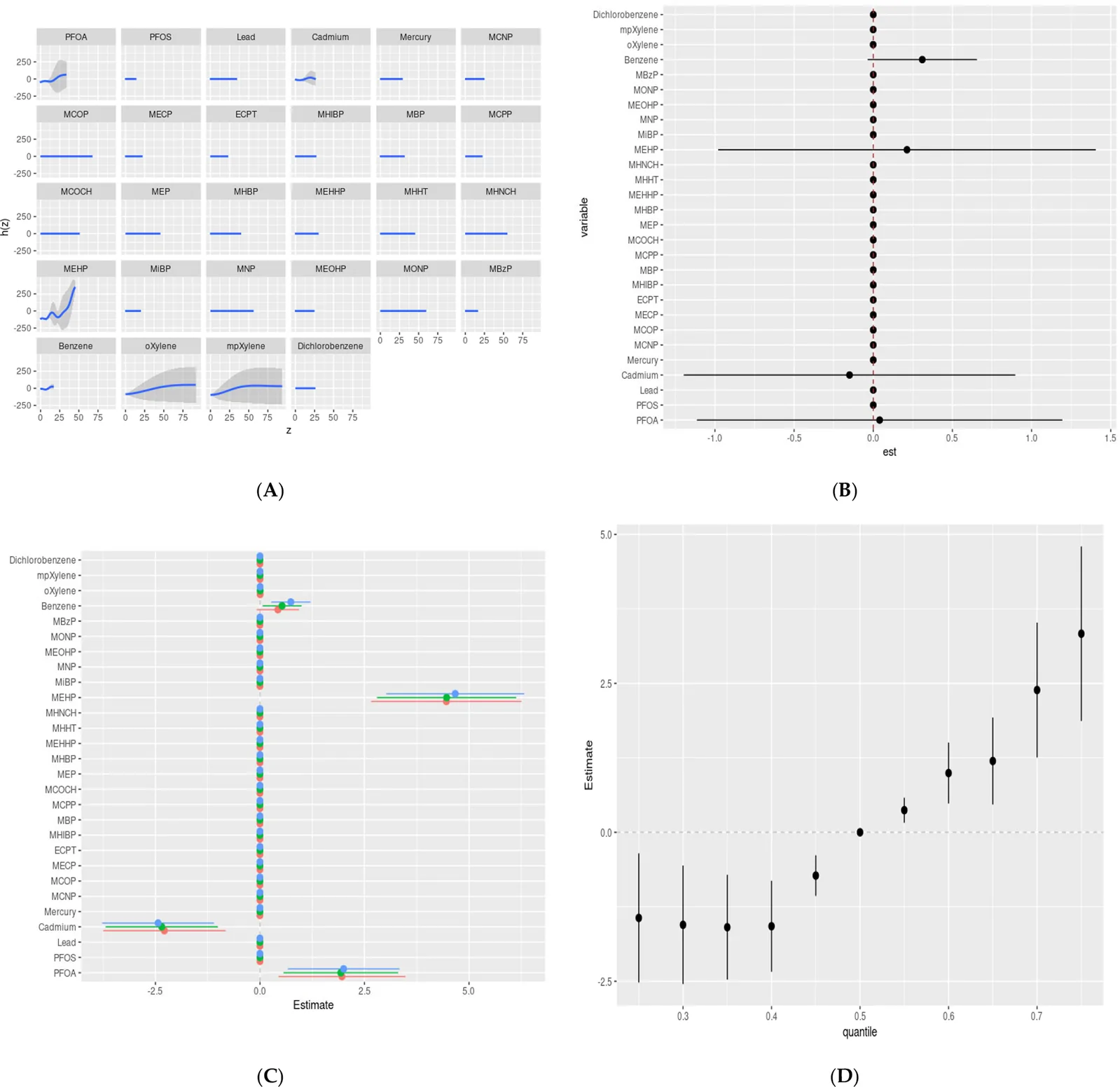
BKMR results for SHBG. (**A**) Univariate exposure–response functions with 95% credible intervals illustrating associations between individual chemical exposures and SHBG concentrations, with other exposures fixed at median values. (**B**) Single-variable summary of the effect of each exposure on SHBG, comparing the 75th to the 25th percentile while all other exposures are fixed at their median. (**C**) Expected change in SHBG associated with increasing each exposure from the 25th to 75th percentile, evaluated when all other exposures are fixed at the 25th (red), 50th (green), or 75th (blue) percentile of the mixture. (**D**) Overall mixture effects and 95% credible intervals for combined chemical exposures on SHBG across mixture quantiles from the 25th to 75th percentiles. Analyses are adjusted for age, sex, race/ethnicity, BMI, smoking, alcohol consumption, diabetes, annual income, hypertension, and steroid use.

**Figure 6.**
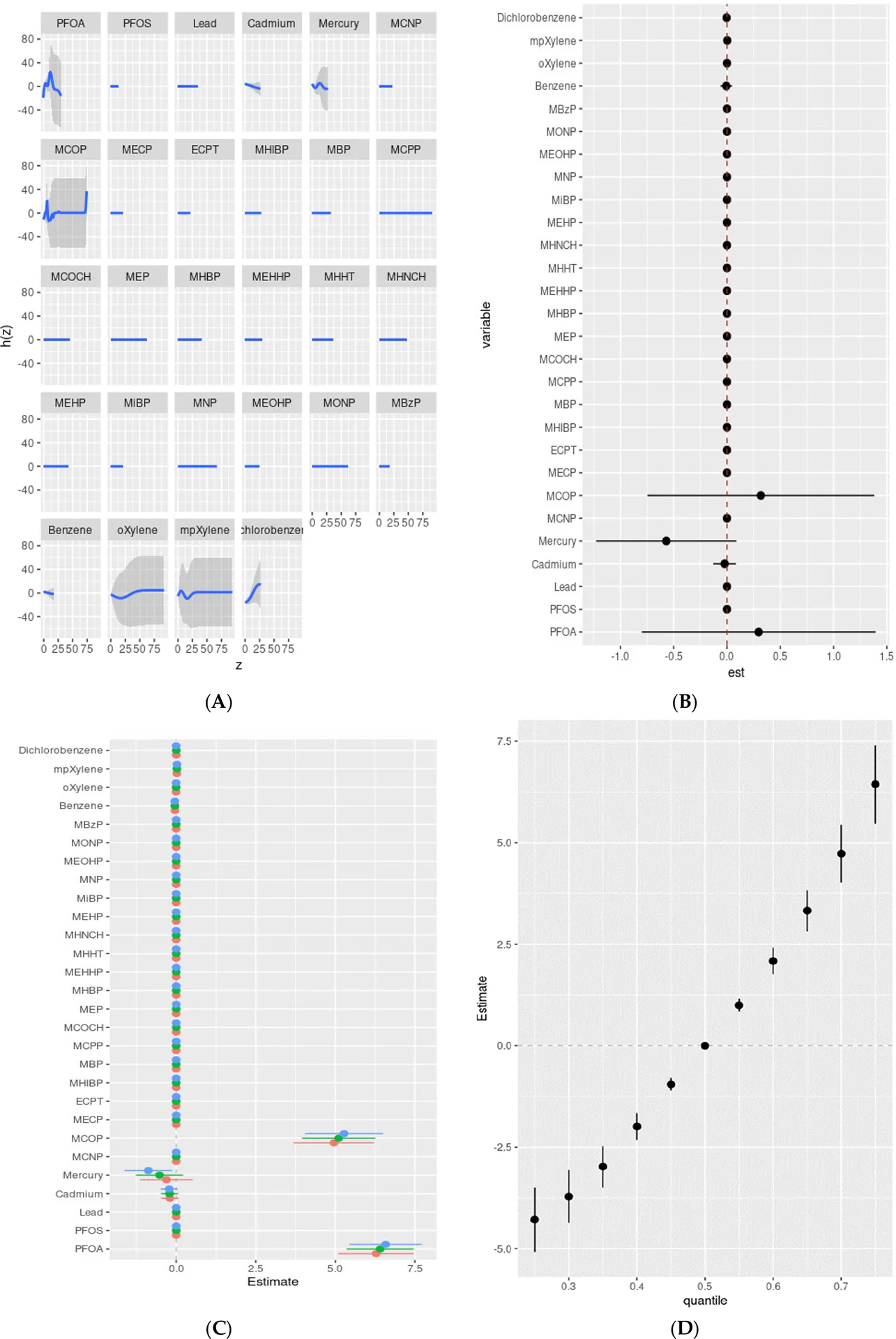
BKMR results for FSH. (**A**) Univariate exposure–response functions with 95% credible intervals illustrating associations between individual chemical exposures and FSH concentrations, with other exposures fixed at median values. (**B**) Single-variable summary of the effect of each exposure on FSH, comparing the 75th to the 25th percentile while all other exposures are fixed at their median. (**C**) Expected change in FSH associated with increasing each exposure from the 25th to 75th percentile, evaluated when all other exposures are fixed at the 25th (red), 50th (green), or 75th (blue) percentile of the mixture. (**D**) Overall mixture effects and 95% credible intervals for combined chemical exposures on FSH across mixture quantiles from the 25th to 75th percentiles. Analyses are adjusted for age, sex, race/ethnicity, BMI, smoking, alcohol consumption, diabetes, annual income, hypertension, and steroid use.

**Figure 7.**
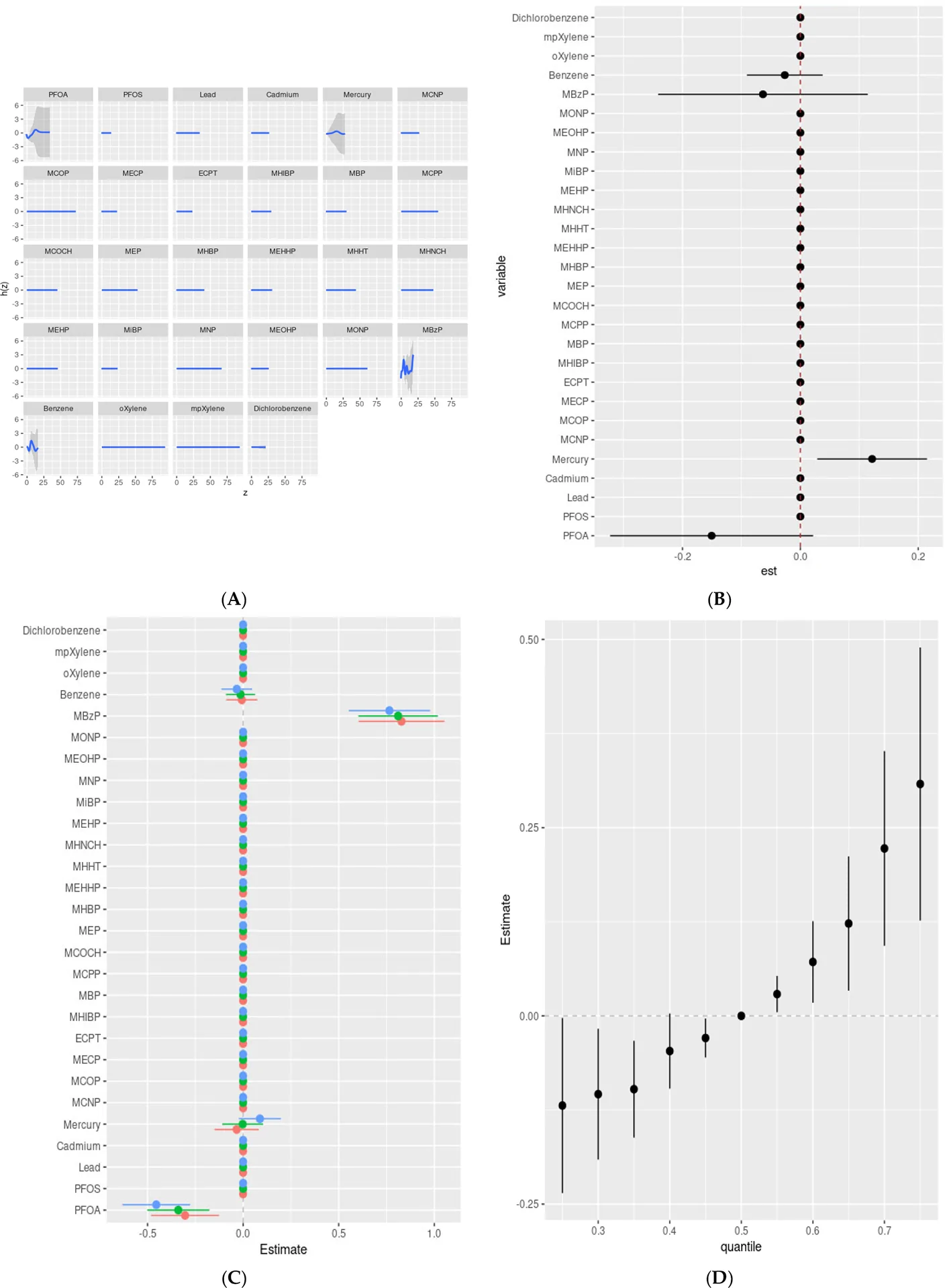
BKMR results for AMH. (**A**) Univariate exposure–response functions with 95% credible intervals illustrating associations between individual chemical exposures and AMH concentrations, with other exposures fixed at median values. (**B**) Single-variable summary of the effect of each exposure on AMH, comparing the 75th to the 25th percentile while all other exposures are fixed at their median. (**C**) Expected change in AMH associated with increasing each exposure from the 25th to 75th percentile, evaluated when all other exposures are fixed at the 25th (red), 50th (green), or 75th (blue) percentile of the mixture. (**D**) Overall mixture effects and 95% credible intervals for combined chemical exposures on AMH across mixture quantiles from the 25th to 75th percentiles. Analyses are adjusted for age, sex, race/ethnicity, BMI, smoking, alcohol consumption, diabetes, annual income, hypertension, and steroid use.

**Figure 8.**
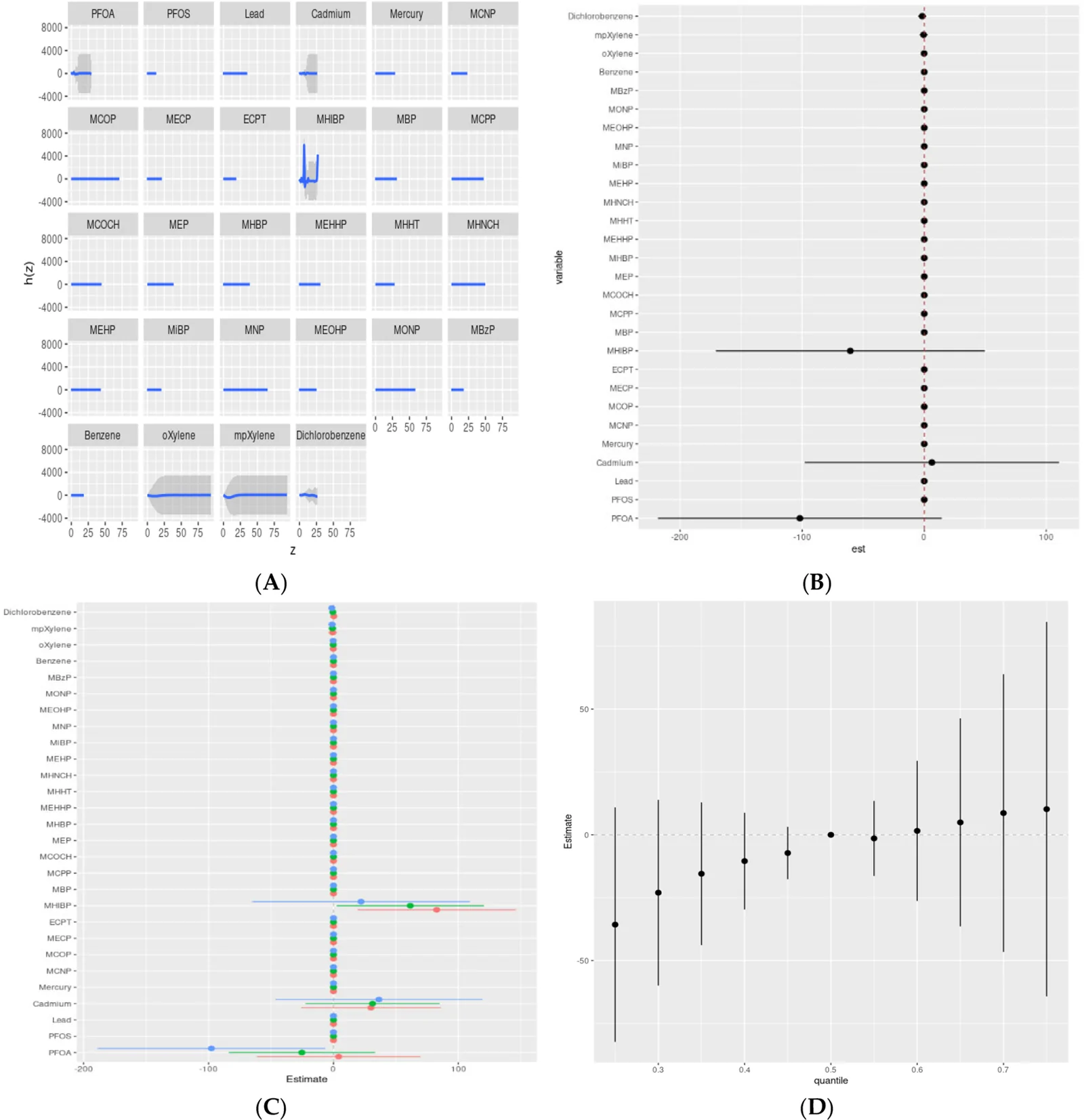
BKMR results for progesterone. (**A**) Univariate exposure–response functions with 95% credible intervals illustrating associations between individual chemical exposures and progesterone concentrations, with other exposures fixed at median values. (**B**) Single-variable summary of the effect of each exposure on progesterone, comparing the 75th to the 25th percentile while all other exposures are fixed at their median. (**C**) Expected change in progesterone associated with increasing each exposure from the 25th to 75th percentile, evaluated when all other exposures are fixed at the 25th (red), 50th (green), or 75th (blue) percentile of the mixture. (**D**) Overall mixture effects and 95% credible intervals for combined chemical exposures on progesterone across mixture quantiles from the 25th to 75th percentiles. Analyses are adjusted for age, sex, race/ethnicity, BMI, smoking, alcohol consumption, diabetes, annual income, hypertension, and steroid use.

**Table 1.** Descriptive statistics for critical variables of the study participants.

Variable	N	Mean	Std. Dev	Minimum	25th Percentile	Median	75th Percentile	Maximum
Age (years)	9254	34.334	25.5	0	11	31	58	80
BMI (kg/m^2^)	8005	26.578	8.261	12.3	20.4	25.8	31.3	86.2
PFOA (ng/mL)	1929	1.71	1.819	0.14	0.87	1.37	2.07	52.87
PFOS (ng/mL)	1929	6.513	7.739	0.14	2.4	4.2	7.8	104.9
Cadmium (μg/L)	7513	0.374	0.503	0.07	0.12	0.22	0.42	13.03
Mercury (μg/L)	7513	1.143	2.27	0.2	0.2	0.51	1.12	63.64
Lead (μg/dL)	6884	1.084	1.291	0.05	0.46	0.76	1.3	42.48
MCNP (ng/mL)	2762	2.441	5.575	0.14	0.7	1.3	2.4	123.5
MCOP (ng/mL)	2762	13.92	76.021	0.21	2.6	5	9.7	3346.1
MECP (ng/mL)	2762	14.793	24.374	0.28	4.6	8.95	16.5	446.9
ECPT (ng/mL)	2762	107.935	304.229	0.14	9.1	27.1	75.4	6066.6
MHIBP (ng/mL)	2762	4.773	10.113	0.28	1.3	2.6	5	249.1
MBP (ng/mL)	2762	16.855	26.041	0.28	5.2	10.8	20	649.1
MCPP (ng/mL)	2762	3.092	42	0.28	0.6	1.1	2.2	2170
MCOCH (ng/mL)	2762	1.802	8.601	0.35	0.35	0.35	1	279.5
MEP (ng/mL)	2762	149	2015.805	0.85	11	26.4	64.7	102,452
MHBP (ng/mL)	2762	1.574	2.776	0.28	0.4	0.9	1.8	82.8
MEHHP (ng/mL)	2762	9.24	15.298	0.28	2.7	5	10.6	371.6
MHHT (ng/mL)	2762	21.82	68.983	0.28	2.4	6	15.8	2346.5
MHNCH (ng/mL)	2762	2.959	14.391	0.28	0.28	0.7	1.6	511
MEHP (ng/mL)	2762	1.824	3.372	0.57	0.57	1	2	115.9
MiBP (ng/mL)	2762	14.673	27.232	0.57	4.2	8.3	16.4	513.6
MNP (ng/mL)	2762	1.098	5.111	0.64	0.64	0.64	0.64	207.7
MEOHP (ng/mL)	2762	6.214	9.657	0.14	1.8	3.75	7	203.3
MONP (ng/mL)	2762	4.05	22.951	0.28	0.7	1.4	2.7	833.3
MBzP (ng/mL)	2762	9.122	18.68	0.21	1.5	3.7	9.2	307.2
Benzene (ng/mL)	2840	0.06	0.129	0.017	0.017	0.017	0.034	2.44
mpXylene (ng/mL)	2856	0.355	12.297	0.024	0.024	0.045	0.084	649
oXylene (ng/mL)	2866	0.078	2.273	0.017	0.017	0.017	0.026	119
Dichlorobenzene (ng/mL)	2855	0.648	5.204	0.028	0.028	0.028	0.088	143
Variable	Description				Frequency (*n*)		Percentage (%)	
					9254			
Sex	Male				4557		49.24	
	Female				4697		50.76	
Race/Ethnicity	Mexican American				1367		14.77	
	Other Hispanics				820		8.86	
	Non-Hispanic white				3150		34.04	
	Non-Hispanic Black				2115		22.85	
	Non-Hispanic Asian				1168		12.62	
	Other, including multiracial				634		6.85	
Alcohol Use					5130			
	Yes				4545		88.6	
	No				585		11.4	
Diabetes					8897			
	Yes				893		10.04	
	No				7816		87.85	
	Borderline				184		2.07	
	Do not know				4		0.04	
Hypertension					1942			
	Yes				1650		84.96	
	No				292		15.04	
Smoking					5856			
	Yes				2359		40.28	
	No				3497		59.72	

MCNP, Mono(carboxynonyl) phthalate; MCOP, Mono(carboxyoctyl) phthalate; MECP, Mono(2-ethyl-5-carboxypentyl) phthalate; MHIBP, Mono(2-hydroxyisobutyl) phthalate; MBP, Mono-n-butyl phthalate; MCPP, Mono(3-carboxypropyl) phthalate; MCOCH, Mono(2-carboxyoctyl) phthalate; MEP, Monoethyl phthalate; MHBP, Mono(2-hydroxy-n-butyl) phthalate; MEHP, Mono(2-ethylhexyl) phthalate; MHHT, Mono(2-ethyl-5-hydroxyhexyl) phthalate; MHNCH, Mono(hydroxy-isononyl) phthalate; MiBP, Mono-isobutyl phthalate; MNP, Mono-n-pentyl phthalate; MEOHP, Mono(2-ethyl-5-oxohexyl) phthalate; MONP, Mono-n-octyl phthalate; MBzP, Monobenzyl phthalate, ECPT, Mono(2-ethyl-5-carboxypentyl) terephthalate.

**Table 2.** Linear regression analysis of exposure variables with the various reproductive hormones.

Variable	Estradiol	FSH	LH	Progesterone	SHBG	AMH
	Coefficient (95%CI)	Coefficient (95%CI)	Coefficient (95%CI)	Coefficient (95%CI)	Coefficient (95%CI)	Coefficient (95%CI)
PFOA	0.933 (−1.629, 3.495)	8.446 (−1.251, 18.143)	6.910 (1.438, 12.382)^†^	−6.297 (−7.602, −4.991)	12.354 (8.332, 16.377)	−1.802 (−2.434, −1.170)
PFOS	−0.017 (−0.634, 0.600)	−0.428 (−2.292, 1.436)	0.080 (−1.002, 1.163)	0.844 (0.540, 1.147)	1.031 (0.119, 1.943)	0.175 (0.048, 0.303)
Cadmium	4.779 (−3.552, 13.109)	26.262 (−0.402, 52.926)	16.182 (1.844, 30.520)^†^	−17.730 (−22.482, −12.979)	−11.973 (−25.768, 1.822)	−3.022 (−4.732, −1.312)
Mercury	−3.383 (−5.115, −1.650)	−0.716 (−6.649, 5.217)	0.296 (−3.125, 3.717)	0.991 (0.136, 1.847)	1.416 (−1.221, 4.052)	0.635 (0.153, 1.117)
Lead	2.542 (1.107, 3.976)	1.949 (−3.034, 6.932)	−0.225 (−2.823, 2.374)	−2.244 (−3.017, −1.472)	−5.229 (−7.640, −2.818)	−0.901 (−1.595, −0.208)
MCNP	1.302 (0.606, 1.999)	−2.323 (−4.400, −0.246)^†^	−1.411 (−2.548, −0.274)^†^	1.175 (0.779, 1.571)	2.566 (1.513, 3.618)	0.307 (0.094, 0.520)
MCOP	0.149 (−0.044, 0.342)	0.091 (−0.680, 0.862)	−0.124 (−0.555, 0.307)	−0.265 (−0.378, −0.153)	−0.928 (−1.240, −0.617)	−0.028 (−0.080, 0.024)
MECP	−0.295 (−0.808, 0.218)	4.093 (2.465, 5.721)	2.451 (1.559, 3.343)	0.370 (0.114, 0.626)	1.419 (0.645, 2.193)	0.003 (−0.191, 0.196)
ECPT	0.036 (−0.015, 0.087)	0.064 (−0.125, 0.253)	0.052 (−0.050, 0.154)	−0.072 (−0.098, −0.046)	−0.412 (−0.494, −0.331)	−0.009 (−0.022, 0.003)
MHIBP	−4.422 (−9.555, 0.712)	22.151 (7.186, 37.116)	12.949 (4.171, 21.727)	−5.210 (−7.911, −2.509)	1.775 (−6.158, 9.709)	−2.588 (−3.595, −1.581)^†^
MBP	0.831 (0.320, 1.343)	−0.753 (−2.726, 1.221)	−0.413 (−1.495, 0.669)	−0.019 (−0.219, 0.181)	−0.178 (−1.045, 0.689)	−0.066 (−0.234, 0.102)
MCPP	−0.814 (−0.981, −0.648)	− 0.372 (−0.959, 0.215)	−0.315 (−0.628, −0.003)^†^	−0.063 (−0.151, 0.025)	−1.356 (−1.635, −1.077)	0.043 (0.006, 0.079)
MCOCH	6.861 (5.704, 8.019)	−11.449 (−15.368, −7.531)	−5.260 (−7.513, −3.006)	− 0.719 (−1.393, −0.045)^†^	−6.828 (−8.237, −5.419)	0.218 (−0.128, 0.565)
MEP	0.005 (−0.029, 0.039)	0.003 (−0.080, 0.086)	0.033 (−0.020, 0.085)	−0.013 (−0.029, 0.002)	−0.095 (−0.135, −0.056)	−0.008 (−0.018, 0.002)
MHBP	−8.719 (−16.027, −1.410)	−9.395 (−36.762, 17.973)	−8.376 (−23.500, 6.748)	2.250 (−0.592, 5.092)	−10.556 (−22.991, 1.879)	2.206 (0.181, 4.232)
MEHHP	1.787 (0.995, 2.579)	− 11.259 (−14.041, −8.477)	−5.014 (−6.531, −3.497)	−4.552 (−6.741, −2.362)	−13.835 (−14.886, −12.783)	0.642 (0.310, 0.973)
MHHT	0.440 (0.117, 0.763)	−1.277 (−2.300, −0.254)^†^	−0.570 (−1.150, 0.010)	0.746 (0.572, 0.919)	2.487 (2.012, 2.961)	0.122 (0.029, 0.216)
MHNCH	−8.443 (−9.818, −7.068)	12.516 (7.384, 17.647)	5.177 (2.391, 7.964)	0.472 (−0.354, 1.298)	7.041 (5.119, 8.963)	−0.423 (−0.847, 0.001)
MEHP	−6.464 (−10.338, −2.590)	16.600 (2.858, 30.341)^†^	7.905 (0.897, 14.914)^†^	−1.225 (−1.619, −0.830)	9.442 (3.080, 15.804)	−1.213 (−2.520, 0.094)
MiBP	−0.005 (−0.910, 0.900)	−2.832 (−6.041, 0.376)	−1.971 (−3.761, −0.181)^†^	0.077 (−0.321, 0.475)	−2.520 (−4.041, −0.999)	0.419 (0.159, 0.680)
MNP	5.657 (−0.443, 11.757)	−20.710 (−35.372, −6.047)	−17.868 (−27.655, −8.080)	−0.873 (−3.677, 1.931)	− 17.448 (−25.366, −9.529)	−0.182 (−2.948, 2.583)
MEOHP	0.683 (−1.458, 2.825)	1.362 (−6.359, 9.083)	−0.400 (−4.505, 3.706)	2.400 (1.460, 3.341)	15.465 (11.776, 19.153)	−0.518 (−1.328, 0.292)
MONP	−1.015 (−2.000, −0.029)^†^	2.426 (−0.636, 5.487)	2.331 (0.538, 4.124)^†^	0.706 (0.184, 1.228)	4.717 (3.156, 6.279)	−0.017 (−0.406, 0.372)
MBzP	0.165 (−0.099, 0.428)	−0.702 (−1.574, 0.171)	−0.158 (−0.580, 0.263)	0.365 (0.212, 0.519)	1.068 (0.591, 1.545)	0.168 (0.080, 0.256)
Benzene	62.005 (1.881, 122.129)^†^	−159.475 (−348.321, 29.371)	−79.993 (−183.744, 23.757)	97.580 (63.590, 131.569)	−61.396 (−158.655, 35.862)	37.038 (20.526, 53.550)
mpXylene	−69.917 (−128.205, −11.629)^†^	180.955 (−60.584, 422.494)	156.421 (29.972, 282.870)^†^	−23.042 (−49.648, 3.563)	524.745 (420.018, 629.472)	−24.534 (−47.927, 1.141)^†^
oXylene	129.468 (−35.163, 294.100)	−497.561 (−1175.809, 180.686)	−423.438 (−778.762, −68.115)^†^	52.118 (−22.846, 127.082)	−1411.399 (−1708.586, −1114.212)	61.712 (−1.597, 125.021)
Dichlorobenzene	0.585 (0.222, 0.947)	−1.180 (−2.232, −0.129)^†^	−0.788 (−1.430, −0.146)	0.442 (0.252, 0.633)	0.706 (0.189, 1.223)	0.213 (0.114, 0.312)

Statistically significant associations are defined as those with *p* < 0.05 (95% CI excludes zero), after adjustment for age, sex, race/ethnicity, BMI, smoking, alcohol consumption, diabetes, annual income, hypertension, and steroid use. Associations that did not remain significant after Benjamini–Hochberg false discovery rate (FDR) correction are marked with ^†^. Regression coefficients for VOC exposures reflect the raw blood concentration scale (ng/mL) and should be interpreted in the context of the interquartile range of each VOC.

**Table 3.** Posterior inclusion probability from hierarchical BKMR results for the influence of each pollutant on the various reproductive hormones.

		Estradiol		FSH		LH		Progesterone		SHBG		AMH	
Variable	Groups	groupPIP	CondPIP	groupPIP	CondPIP	groupPIP	CondPIP	groupPIP	CondPIP	groupPIP	CondPIP	groupPIP	CondPIP
PFOA	1	1.0000	0.0000	1.0000	1.0000	0.0700	0.7600	1.0000	1.0000	0.6072	1.0000	1.0000	1.0000
PFOS	1	1.0000	1.0000	1.0000	0.0000	0.0700	0.2400	1.0000	0.0000	0.6072	0.0000	1.0000	0.0000
Cadmium	2	1.0000	1.0000	1.0000	0.0000	0.8764	0.9772	1.0000	0.0000	0.5596	0.0000	1.0000	0.0000
Mercury	2	1.0000	0.0000	1.0000	0.0044	0.8764	0.0000	1.0000	1.0000	0.5596	1.0000	1.0000	0.0000
Lead	2	1.0000	0.0000	1.0000	0.9956	0.8764	0.0228	1.0000	0.0000	0.5596	0.0000	1.0000	1.0000
MCNP	3	1.0000	0.0000	1.0000	0.0000	1.0000	0.0000	1.0000	0.0000	1.0000	0.0000	1.0000	0.0000
MCOP	3	1.0000	0.0000	1.0000	0.0000	1.0000	0.0000	1.0000	0.0000	1.0000	0.0000	1.0000	0.0000
MECP	3	1.0000	0.0000	1.0000	0.0000	1.0000	0.0000	1.0000	0.0000	1.0000	0.0000	1.0000	0.0000
ECPT	3	1.0000	0.0000	1.0000	0.0000	1.0000	0.0000	1.0000	0.0000	1.0000	0.0000	1.0000	0.0000
MHIBP	3	1.0000	0.0000	1.0000	0.0000	1.0000	0.0000	1.0000	1.0000	1.0000	0.0000	1.0000	0.0000
MBP	3	1.0000	0.0000	1.0000	0.0000	1.0000	0.0000	1.0000	0.0000	1.0000	0.0000	1.0000	0.0000
MCPP	3	1.0000	0.0000	1.0000	0.0000	1.0000	0.0000	1.0000	0.0000	1.0000	0.0000	1.0000	0.0000
MCOCH	3	1.0000	0.0000	1.0000	0.0000	1.0000	0.0000	1.0000	0.0000	1.0000	0.0000	1.0000	0.0000
MEP	3	1.0000	0.0000	1.0000	0.0000	1.0000	0.0000	1.0000	0.0000	1.0000	0.0000	1.0000	0.0000
MHBP	3	1.0000	0.0000	1.0000	0.0000	1.0000	0.0000	1.0000	0.0000	1.0000	0.0000	1.0000	0.0000
MEHHP	3	1.0000	0.0000	1.0000	0.0000	1.0000	0.0000	1.0000	0.0000	1.0000	0.0000	1.0000	0.0000
MHHT	3	1.0000	0.0000	1.0000	0.0000	1.0000	0.0000	1.0000	0.0000	1.0000	0.0000	1.0000	0.0000
MHNCH	3	1.0000	0.0000	1.0000	0.0000	1.0000	0.0000	1.0000	0.0000	1.0000	0.0000	1.0000	0.0000
MEHP	3	1.0000	0.0000	1.0000	0.0000	1.0000	0.0000	1.0000	0.0000	1.0000	1.0000	1.0000	0.0000
MiBP	3	1.0000	0.0000	1.0000	0.0000	1.0000	0.0000	1.0000	0.0000	1.0000	0.0000	1.0000	0.0000
MNP	3	1.0000	0.0000	1.0000	0.0000	1.0000	0.0000	1.0000	0.0000	1.0000	0.0000	1.0000	0.0000
MEOHP	3	1.0000	0.0000	1.0000	0.0000	1.0000	0.0000	1.0000	0.0000	1.0000	0.0000	1.0000	0.0000
MONP	3	1.0000	0.0000	1.0000	0.0000	1.0000	0.0000	1.0000	0.0000	1.0000	0.0000	1.0000	0.0000
MBzP	3	1.0000	1.0000	1.0000	0.0000	1.0000	1.0000	1.0000	0.0000	1.0000	0.0000	1.0000	1.0000
Benzene	4	1.0000	0.0912	0.6396	0.0419	1.0000	0.0000	0.9016	0.0000	0.5040	0.9333	1.0000	0.9996
mpXylene	4	1.0000	0.9088	0.6396	0.1676	1.0000	1.0000	0.9016	0.1637	0.5040	0.0222	1.0000	0.0000
oXylene	4	1.0000	0.0000	0.6396	0.5210	1.0000	0.0000	0.9016	0.1167	0.5040	0.0444	1.0000	0.0000
Dichlorobenzene	4	1.0000	0.0000	0.6396	0.2695	1.0000	0.0000	0.9016	0.7196	0.5040	0.0000	1.0000	0.0004

## Data Availability

The data presented in this study are openly available on the CDC NHANES site at https://wwwn.cdc.gov/nchs/nhanes/continuousnhanes/overview.aspx?BeginYear=2017 (accessed on 18 February 2026).
